# Supplementary material for: Standard operating procedure combined with comprehensive quality control system for multiple LC-MS platforms urinary proteomics
Source: Nat Commun. 2025 Jan 26;16:1051. doi: 10.1038/s41467-025-56337-4 (PMC11770173; doi:10.1038/s41467-025-56337-4)
Supplement: Supplementary file 1 — Supplementary Information [file 41467_2025_56337_MOESM1_ESM.pdf]

Supplementary Information for:
Standard operating procedure combined with comprehensive quality control system for multiple LC-MS platforms urinary proteomics

Xiang Liu[1,#], Haidan Sun[2,#], Xinhang Hou[1,#], Jiameng Sun[2], Min Tang[1], Yong-biao Zhang[1], Yongqian Zhang[3], Wei Sun[2,*], Chao Liu[1,*] & Urine Test Sample Working Group[4]

# These authors contributed equally to this work
* To whom correspondence should be addressed:
C.L. (liuchaobuaa@buaa.edu.cn), W.S. (sunwei@ibms.pumc.edu.cn)

1. School of Biological Science and Medical Engineering & School of Engineering Medicine, Beihang University, Beijing, China.
2. Proteomics Center, Core Facility of Instrument, Institute of Basic Medical Sciences Chinese Academy of Medical Sciences, School of Basic Medicine Peking Union Medical College, Beijing, China.
3. School of Medical Technology, Beijing Institute of Technology, Beijing, China.
4. A full list of authors appears at the end of this paper.

Contents

Supplementary Figure 1. The overview of MSCohort intra-experiment analysis for DIA experiment. 4

Supplementary Figure 2. The overview of MSCohort inter-experiment analysis. 5

Supplementary Figure 3. Heatmap of intra-experiment metrics score for data from 20 LC-MS platforms and the comparison of performance-associated metrics in 8 Orbitrap data without LC-SOP. 6

Supplementary Figure 4. The numbers of proteins co-identified across various combinations of 6 platforms without LC-SOP. 7

Supplementary Figure 5. The analysis of the protein sequence coverage, the relative abundance, and the dynamic range across 20 LC-MS platforms. 8

Supplementary Figure 6. The summary of qualification and quantification results from 3 groups, which divided based on the data collection conditions. 9

Supplementary Figure 8. Overall quantitative performance of sample A-H and sample B-H from 3 LC-MS platforms. 11

Supplementary Figure 9. Quality assessment for clinical colorectal cancer urinary proteomics experiments. 12

Supplementary Figure 10. Clinical colorectal cancer urinary proteomics analysis in three LC-MS platforms. 13

Supplementary Figure 11. GO enrichment analysis and KEGG pathway analysis of urine DEPs between CRC and HC from 3 LC-MS platform and tissue DEPs between CRC and HC from previous study. 14

Supplementary Figure 12. Protein-protein interaction network associated with colorectal cancer (CRC). 15

Supplementary Figure 13. Generation of the CRC urinary protein biomarker signature. 16

Supplementary Figure 14. The retention time and peak width of pure iRT peptides from 20 LC-MS platforms. 17

Supplementary Figure 15. The qualitative and quantitative performance of DDA data across 20 LC-MS platforms. 18

Supplementary Figure 16. The quality assessment of carry-over for LC system for 20 LC-MS platforms 19

Supplementary Note 1: Comparison of the Metrics in MSCohort and the Existing Metrics in other Quality Control Tools 20

Supplementary Note 2: Comparison of MSCohort and other Quality Control Tools 26

Supplementary Note 3: The Standard Operating Procedure (SOP) for Urinary Proteomics 29

Supplementary Note 4: Optimization of LC-MS Method for Establishment of SOP for Urinary Proteomics 44

Supplementary Note 5: The Novelty of Our Developed Standard Operating Procedure (SOP) for Urinary Proteomics 53

Supplementary Note 6: Comparative analysis of urine proteome DDA data from multi-platform study 55

Supplementary Methods 56

References 58

Supplementary Figure 1. The overview of MSCohort intra-experiment analysis for DIA experiment.

(a) Metric−score diagram for intra-experiment analysis. The LC−MS workflow includes multiple procedures: sample preparation, liquid chromatography, and mass spectrometry. Dozens of metrics and variations in these procedures ultimately affect the number of acquired MS2 scans ($N_{acquired\_MS2}$), the identification rate of MS2 scans (Q_{MS2}), the spectra complexity of MS2 scans ($N_{precursor\_per\_MS2}$), and the precursors duplicate identification rate ($R_{precursor}$). **(b)** Firstly, the paths of raw files and identification results can be entered by users, and then MSCohort reads these files and calculates several second-level scores and five first-level scores. The average of the five first-level scores is the total score of the data. Finally, MSCohort outputs a report of the LC−MS experiment including scores and some other tables and figures.

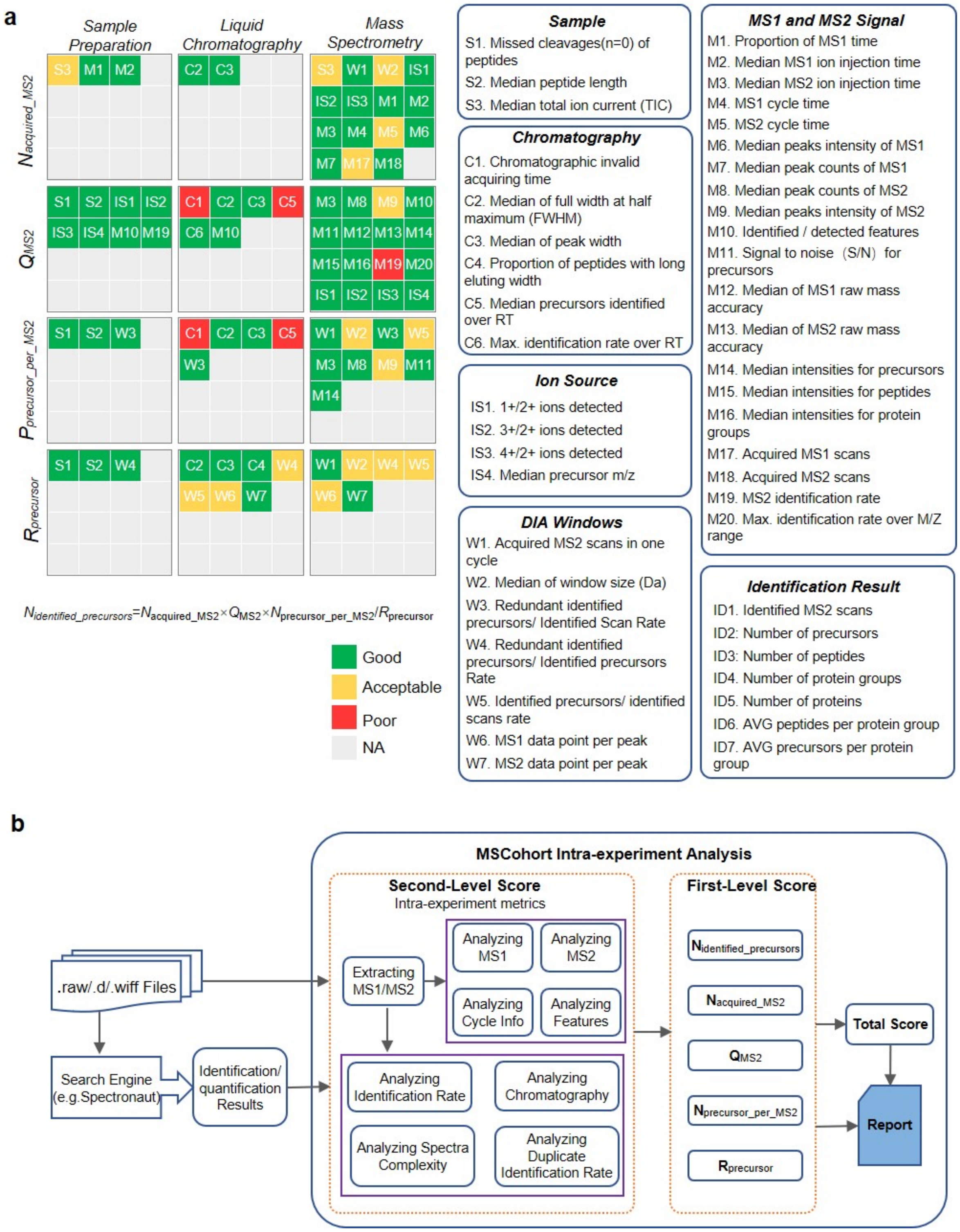

Supplementary Figure 2. The overview of MSCohort inter-experiment analysis.

Metric−score diagram for inter-experiment analysis. Inter-experiment module extracts 23 metrics in precursor, peptide, and protein groups levels mapping to LC−MS workflow, to facilitate the comprehensive quality evaluation for cohort data, flag low-quality data, and correct data heterogeneity for subsequent analyses.

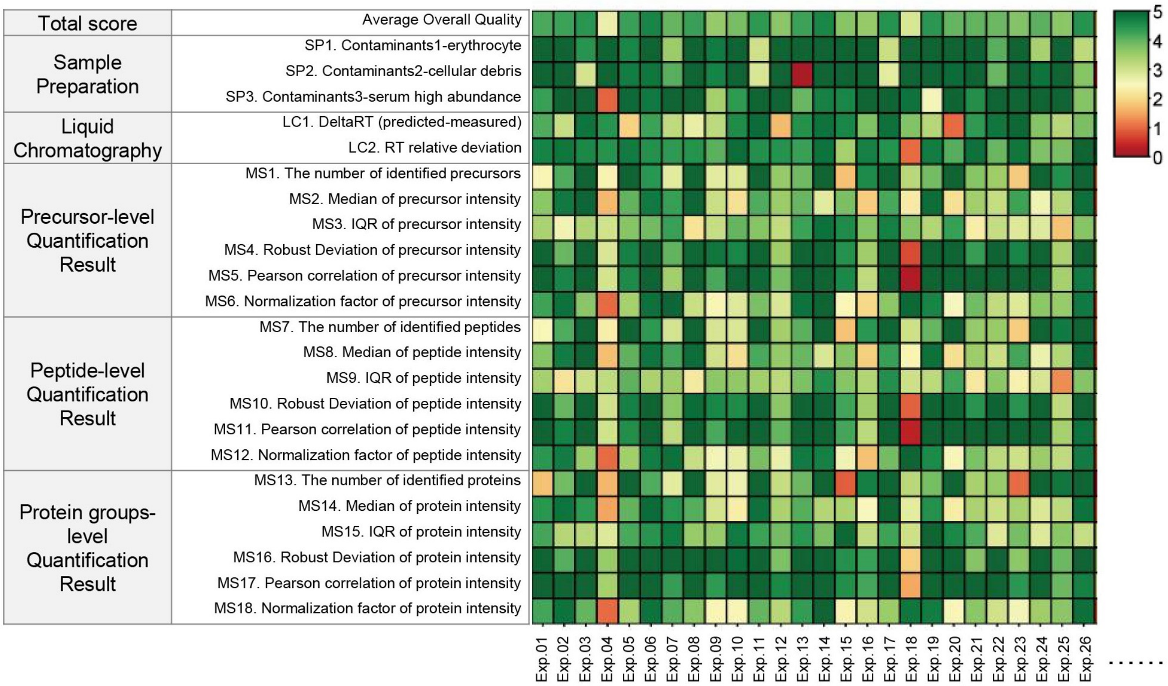

Supplementary Figure 3. Heatmap of intra-experiment metrics score for data from 20 LC-MS platforms and the comparison of performance-associated metrics in 8 Orbitrap data without LC-SOP.

(a) Heatmap representation of metrics score from 20 LC-MS platforms. See Supplementary Data 4 for the summary of metrics values and metrics scores. The four platforms showed a lower identification result and the lower score metrics were placed in red font. **(b)** The number of acquired MS2 scans, identified precursors, and identified peptides in 8 Orbitrap platforms. Horizontal dashed lines were added at 15000, as a reference value. **(c)** The overall identification rate (ID rate) of MS2 scans in 8 Orbitrap platforms. The color of the bar chart represented the score for this metric in (a). **(d)** The utilization of MS2 scans ($N_{precursor\_per\_MS2}$ / $R_{precursor}$) in 8 Orbitrap platforms. The spectral complexity ($N_{precursor\_per\_MS2}$) was greater than the precursors duplicate identification rate ($R_{precursor}$), resulting in higher utilization of MS2 scans. **(e)** The distribution of log10 MS2 peaks intensity in one scan in 8 Orbitrap platforms. The three lines represented the 1st quartile, the median, and 3rd quartile, respectively. The color of the bar chart represented the score for this metric in (a). **(f)** The distribution of MS2 peak counts in one scan in 8 Orbitrap platforms. The three lines represented the 1st quartile, the median, and 3rd quartile, respectively. The color of the bar chart represented the score for this metric in (a). **(g)** The median peak width and full width at half maximum (FWHM) in 8 Orbitrap platforms. **(h)** The invalid acquiring time in 8 Orbitrap platforms. The color of the bar chart represented the score for this metric in (a). **(i)** The distribution of MS2 ion injection time in 8 Orbitrap platforms. The color of the bar chart represented the score for this metric in (a).

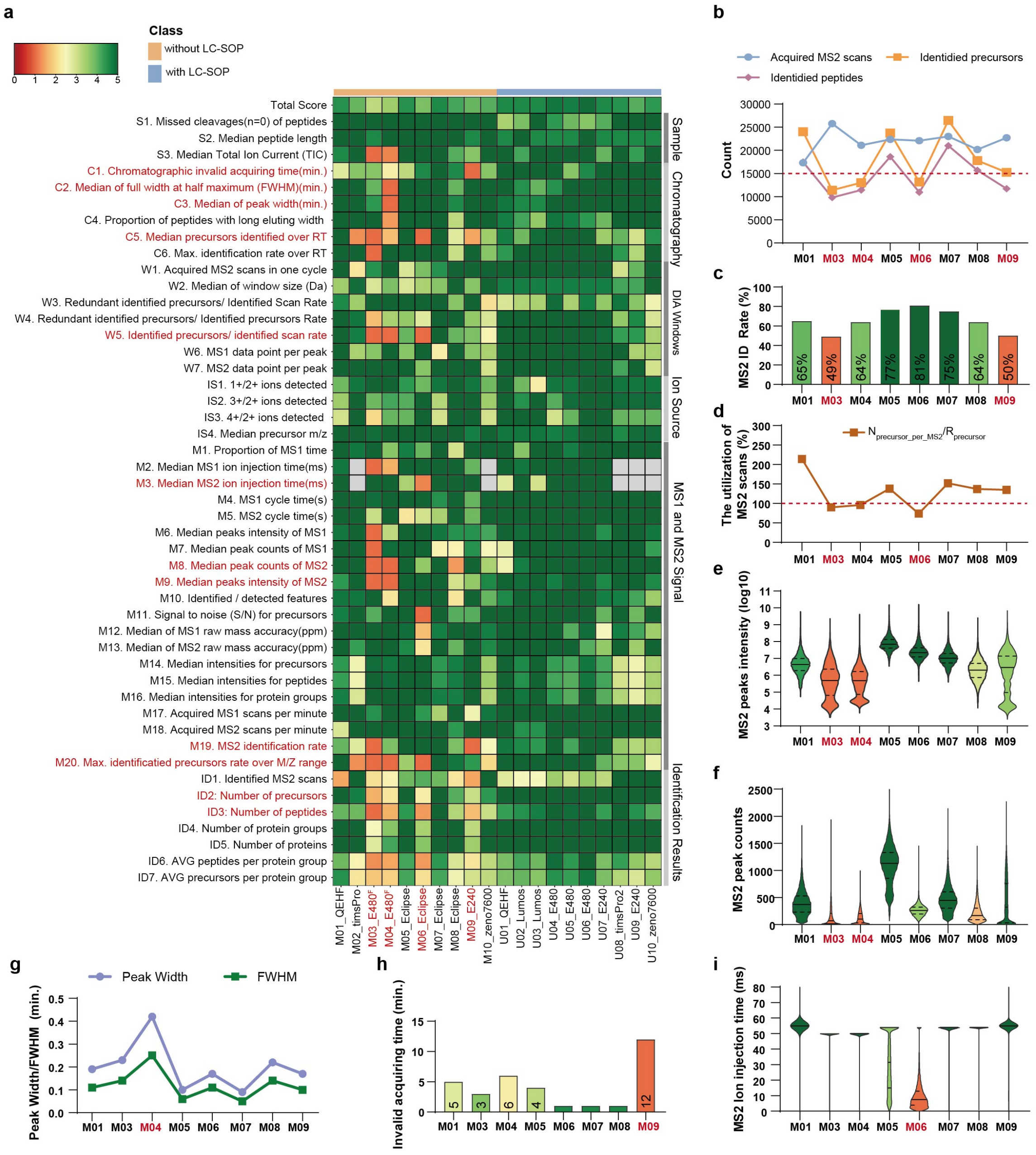

Supplementary Figure 4. The numbers of proteins co-identified across various combinations of 6 platforms without LC-SOP.

The vertical green bar (2876 proteins) reflects the number of proteins co-identified by 6 platforms (M03, M04, M06, M09 are not included in the group). The horizontal bars reflect the number of proteins identified in total at each platform.

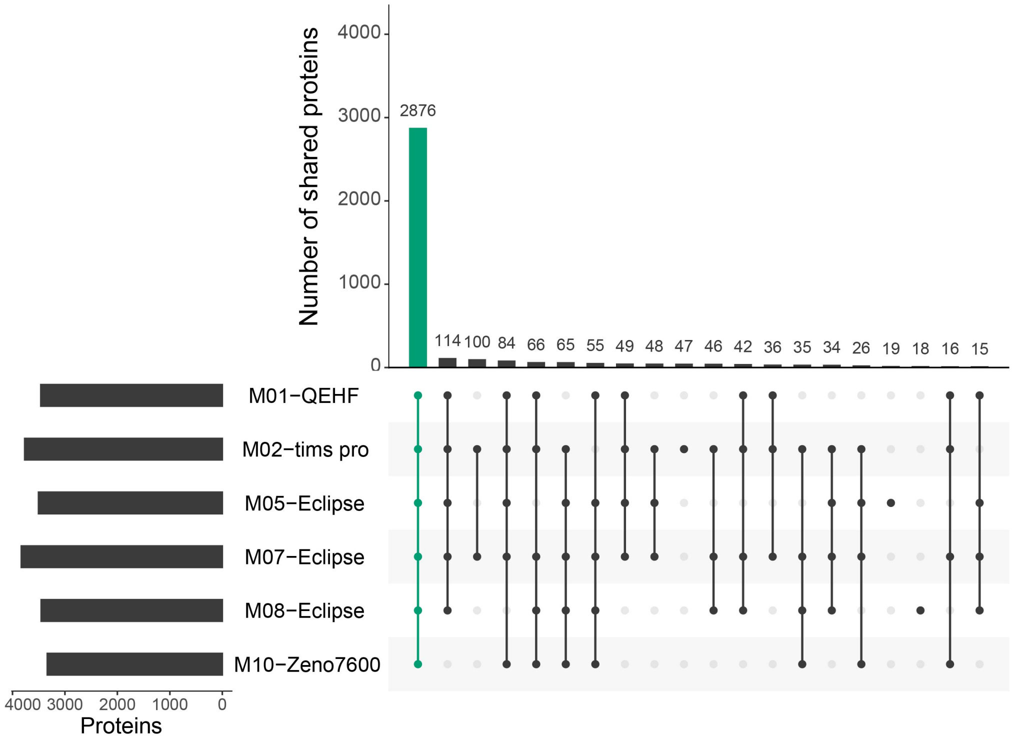

Supplementary Figure 5. The analysis of the protein sequence coverage, the relative abundance, and the dynamic range across 20 LC-MS platforms.

(a) and **(b)** The protein sequence coverage for the 10 platforms without LC-SOP (a, orange) and with LC-SOP (b, blue). The vertical black line represents the median of protein sequence coverage, and the median value was listed. **(c)** and **(d)** The relative abundance of Log 10 protein intensity for the 10 platforms without LC-SOP (c, orange) and with LC-SOP (d, blue). **(e)** and **(f)** The dynamic range of quantified protein intensity for the 10 platforms without LC-SOP and with LC-SOP.

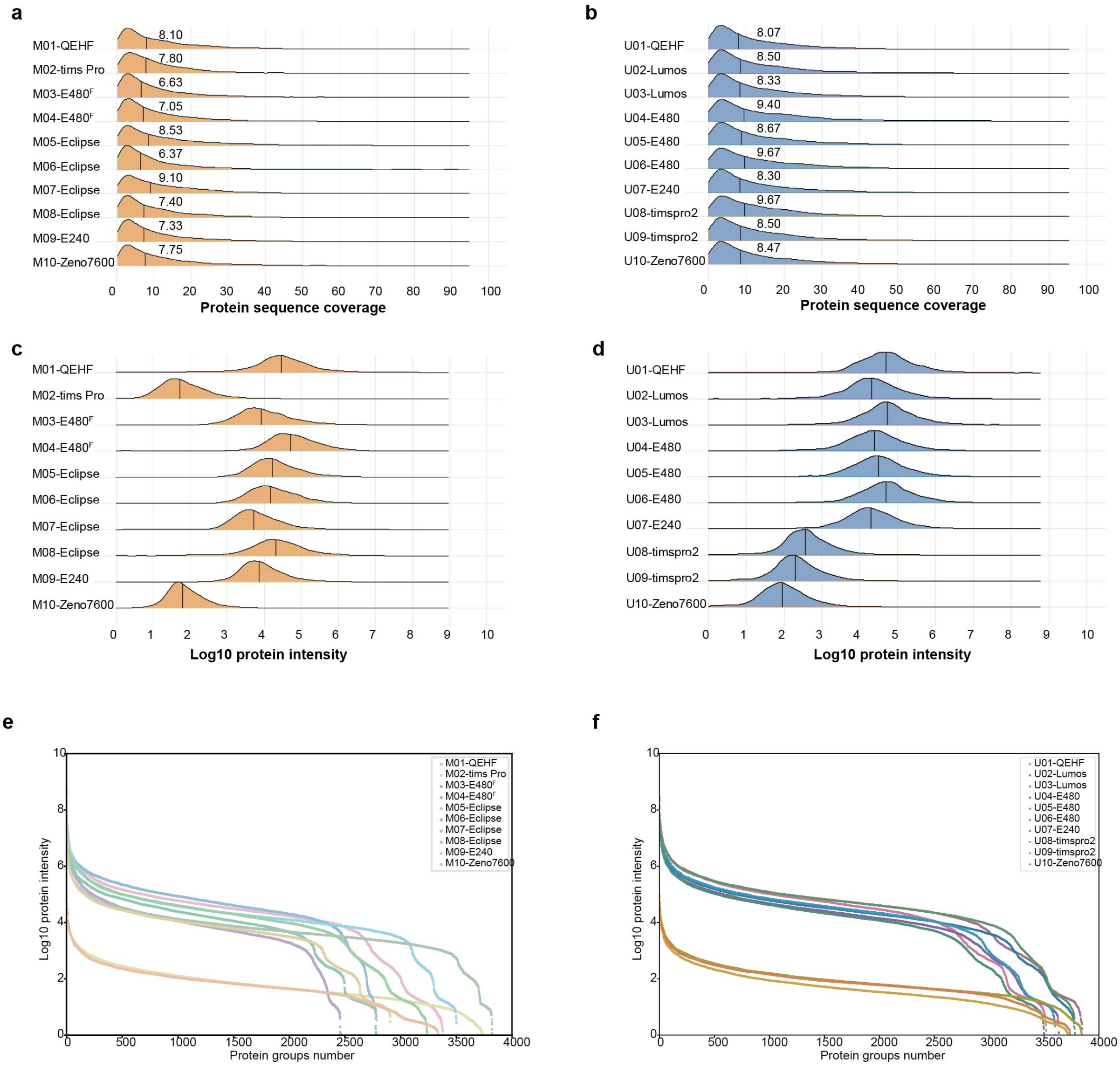

Supplementary Figure 6. The summary of qualification and quantification results from 3 groups, which divided based on the data collection conditions.

(a) The data from the 11 Orbitrap platforms were divided into 3 groups according to LC and MS conditions (M03, M04, M06, M09 are not included in the group), and the analysis of qualitative and quantitative results were summarized. **(b)** The number of identified protein groups, peptides, and precursors in each group. Bars represented the mean values of the group, the mean number was shown in the bottom of the bar, and error bars represented +/− SD. **(c)** The distribution of the coefficients of variation (CV) obtained on protein/peptide/precursor intensity from each group. The red line represented the median, and the blank lines represented the first and third quartiles. The median CVs were placed in red font. **(d)** The distribution of the Pearson correlation coefficients obtained from each group. The solid line represented the median, and error bars representing +/− SD. The median of r was placed in red font.

a

	Group	Acquisition methods	Dataset	Number of ID range	Mean number of IDs	SD (Standard Deviation)	RSD (relative standard deviation)	Median of Pearson r (range)	Quant Median CV (coefficient of variation)
Protein Groups	A	Same MS condition, same LC condition	U04, U05, U06	3400-3696	3572	±109.5	3.00%	0.96 (0.95-0.97)	0.28
	B	Different MS condition, same LC condition	U01, U02, U03, U07	3335-3533	3404	±63.2	1.80%	0.95 (0.92-0.96)	0.36
	A+B	Different MS condition, same LC condition	U01, U02, U03, U04, U05, U06, U07	3335-3696	3476	±119.48	3.43%	0.95 (0.92-0.97)	0.40
	C	Different MS condition, different LC condition	M01, M05, M07, M08	3128-3698	3328	±221.4	6.70%	0.95 (0.91-0.96)	0.41
Peptides	A	Same MS condition, same LC condition	U04, U05, U06	19477-22994	21373	±1307.7	6.12%	0.92 (0.91-0.93)	0.42
	B	Different MS condition, same LC condition	U01, U02, U03, U07	17891-19296	18527	±377.6	2.04%	0.88 (0.84-0.92)	0.52
	A+B	Different MS condition, same LC condition	U01, U02, U03, U04, U05, U06, U07	17891-22994	19747	±1672.6	8.47%	0.9 (0.84-0.93)	0.60
	C	Different MS condition, different LC condition	M01, M05, M07, M08	15005-21058	17973	±2115.8	11.80%	0.85 (0.83-0.88)	0.68
Precursors	A	Same MS condition, same LC condition	U04, U05, U06	24729-29013	27022	±1519.1	5.60%	0.93 (0.91-0.95)	0.42
	B	Different MS condition, same LC condition	U01, U02, U03, U07	22556-23718	23208	±317.9	1.40%	0.90 (0.86-0.94)	0.53
	A+B	Different MS condition, same LC condition	U01, U02, U03, U04, U05, U06, U07	22556-29013	24843	±2146.9	8.64%	0.92 (0.86-0.95)	0.60
	C	Different MS condition, different LC condition	M01, M05, M07, M08	16887-26615	22704	±3368.1	14.80%	0.88 (0.84-0.92)	0.65

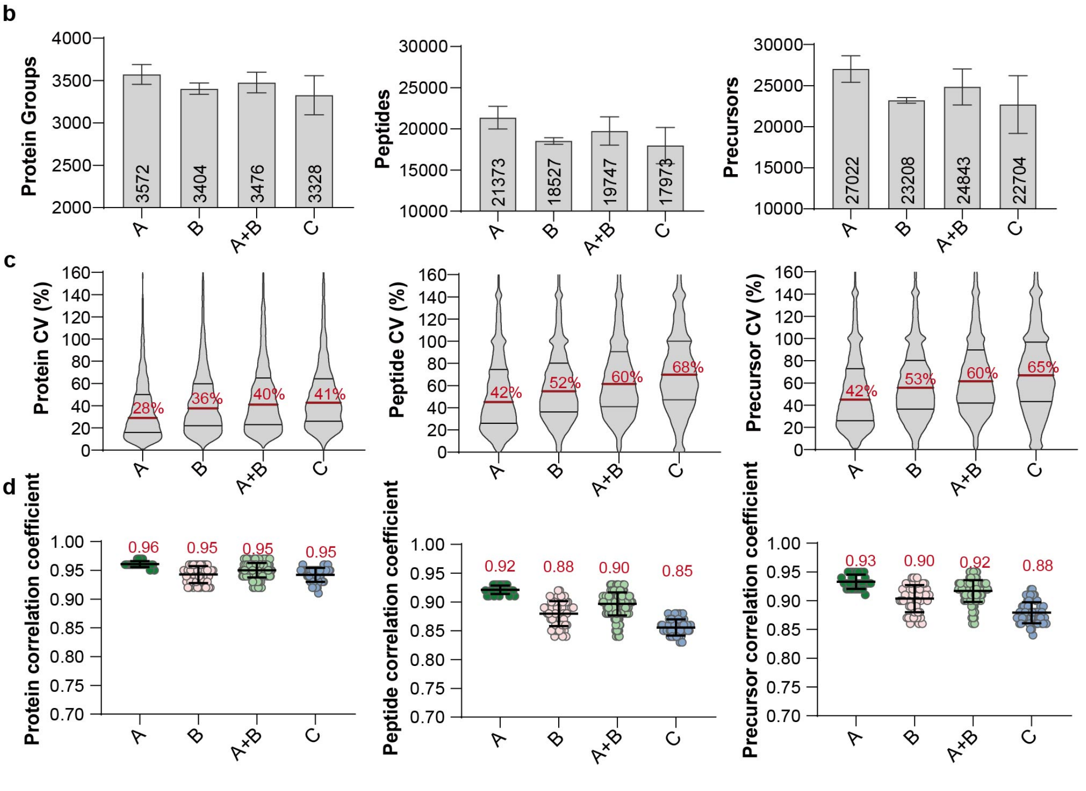

Supplementary Figure 7. Overall qualitative and quantitative performance of benchmarking samples from 3 LC-MS platforms.

(**a**) The number of proteins identified from sample-A and sample-B in Lumos, E480, and TIMS, respectively. (**b**) The distribution of the CV obtained on the quantified protein intensity across the three technical replicates was plotted for 3 LC-MS platforms. Boxplots with the center line, box bounds, and whiskers represent the median, 1st/3rd quartile, and 10th/90th percentile, respectively. Horizontal dashed lines were added at 20% CV, as a reference value. (**c**) Overlap of total identified protein groups from Lumos, E480, and TIMS. (**d**) The Pearson correlation matrix based on log2 protein intensity of the common proteins from 3 LC-MS platforms. The color-scale indicates the magnitude of the Pearson correlation coefficient.

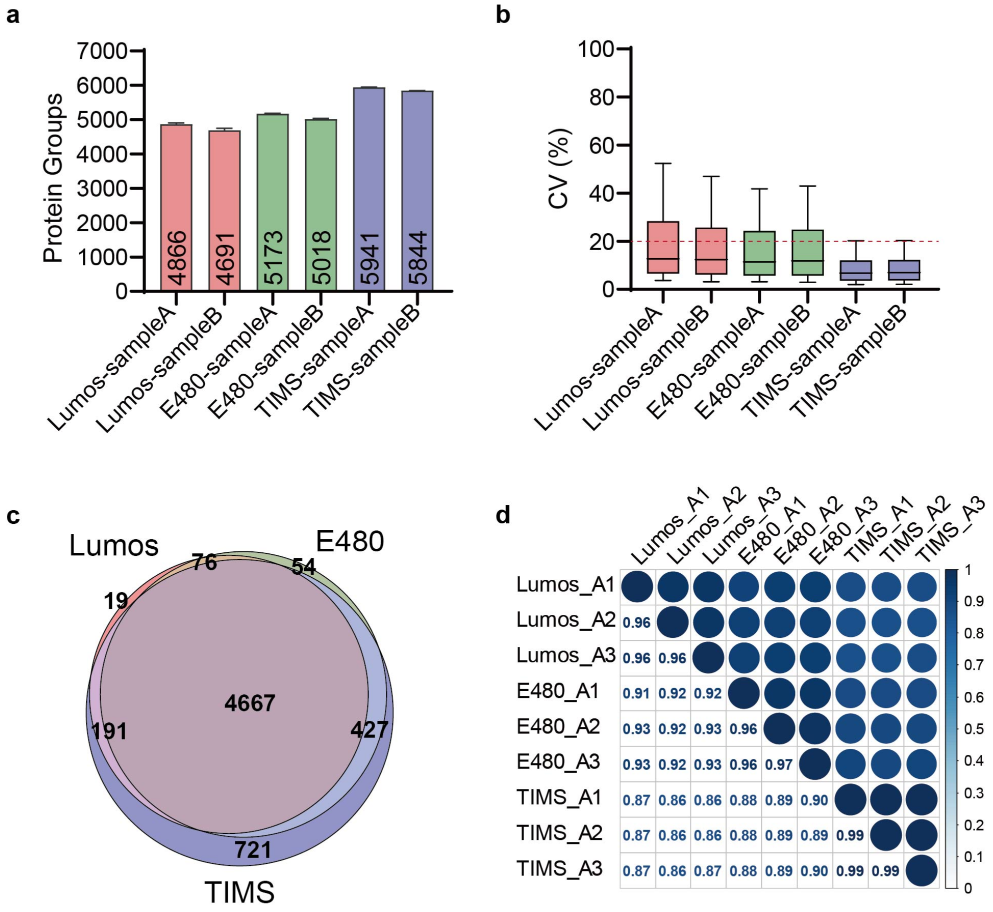

Supplementary Figure 8. Overall quantitative performance of sample A-H and sample B-H from 3 LC-MS platforms.

Benchmarking samples A-H and B-H were prepared containing known ratios of peptide digestions from human HEK 293 cell, yeast and E. coli, resulting in expected peptide and protein ratios of 1:1 (A/B) for human, 1:2 for yeast and 4:1 for *E. coli* proteins. **(a)** The number of proteins identified for each individual organism from sample A-H and sample B-H in Lumos, E480, and TIMS, respectively. **(b)** Log-transformed ratios (log2(A/B)) of proteins plotted over the log-transformed intensity of sample B in Lumos, E480, and TIMS (proteins). Colored dashed lines represented the expected log2(A/B) values for human (green), yeast (orange), and *E. coli* (purple) proteins. All box plots indicated the median and the first and third quartiles as the box ends. Whiskers were positioned 1.5-times the interquartile range. **(c)** The distribution of the CV obtained on the quantified protein intensity across the three technical replicates was plotted for each individual organism. All box plots indicated the median and the first and third quartiles as the box ends. Whiskers were positioned 1.5-times the interquartile range. **(d)** The relative deviation from theoretical ratio for three organisms from Lumos, E480, and TIMS, respectively. **(e)** Differentially expressed protein (DEP) detection for three organisms from Lumos, E480, and TIMS, respectively. Percentages of significantly changed proteins as DEPs over the total number of quantified proteins in 1:2 and 4:1 conditions were used to estimate the sensitivity, while those in 1:1 conditions were used to estimate the specificity. **(f)** Sensitivity and specificity of the DEP analysis based on receiver operating characteristic (ROC) curves.

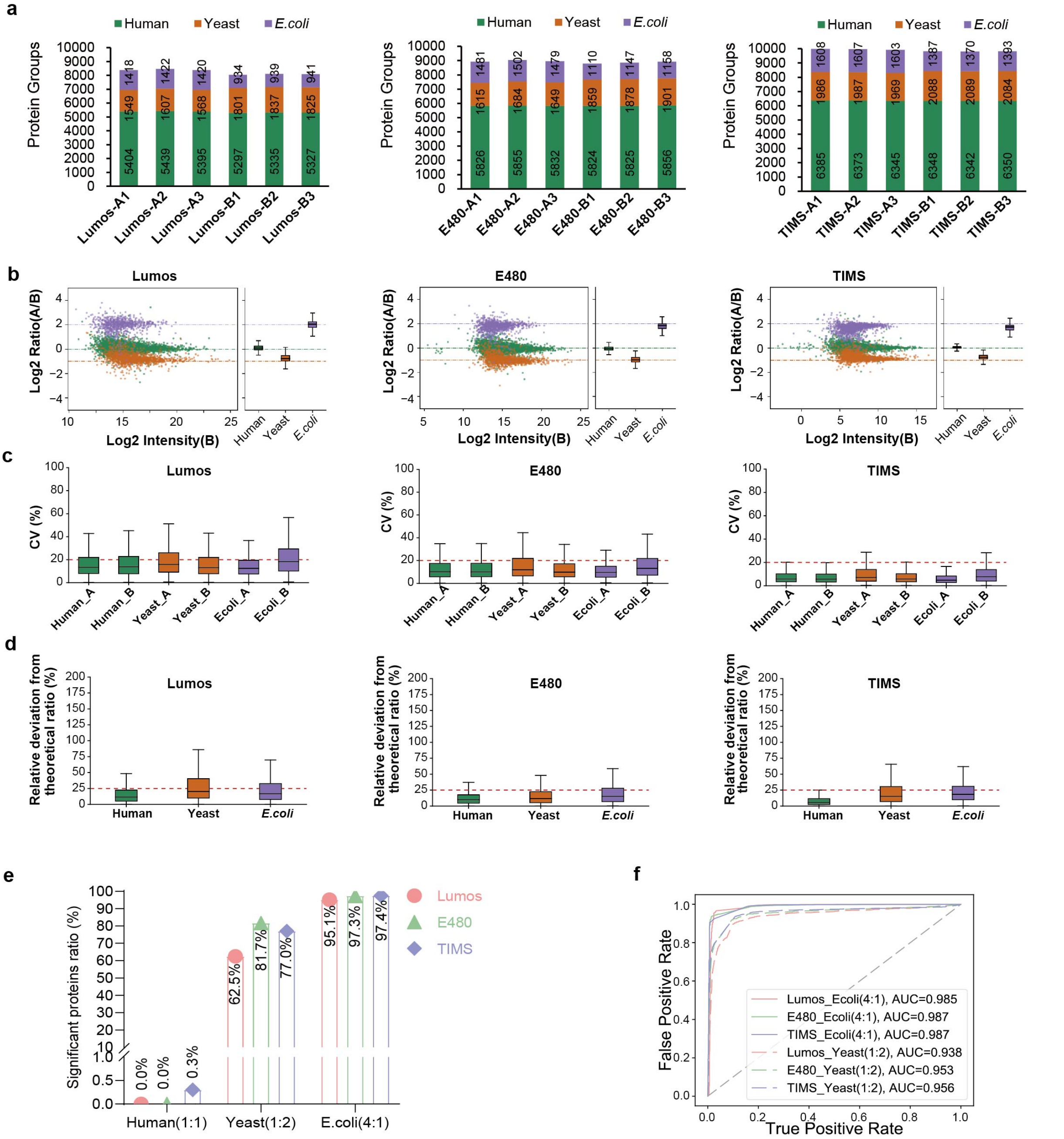

Supplementary Figure 9. Quality assessment for clinical colorectal cancer urinary proteomics experiments.

(a) Pearson correlation between proteomics experiments of the quality control (QC) sample replicates in Lumos, E480, and TIMS, respectively. **(b)** The anomaly score plot of the LC-MS experiments in TIMS. Experiments identified as outliers (red marks) sit under the grey horizontal line which represents the experiment score below the critical value (-0.05). The anomaly score plot of the LC-MS experiments in Lumos and E480 showed in Supplementary Data 3. **(c)** Metric−score diagram for inter-experiment analysis in TIMS. Rows denoted the metrics; columns corresponded to LC-MS experiments. The color-scale indicated the magnitude of the inter-experiment score. The eight low-quality experiments indicated by the red arrows showed a low score (at least 7 of 23 metrics showed a variation more than two standard deviations (SD) from its median). **(d)** Assessment of individual experiment quality with respect to the three contamination quality marker panels. The horizontal yellow line and red lines represents that are more than two standard deviations and three standard deviations from the median, respectively. The experiments indicated by the red arrows showed the performance of eight low-quality experiments in contaminants metrics. **(e)** Pearson correlation coefficient of quantitative intensity between the 160 samples. The experiments indicated by the red arrows showed a low correlation with other experiments. **(f)** The robust standard deviations of intensity ratio between pairs of experiments. The experiments indicated by the red arrows showed a high standard deviation with other experiments.

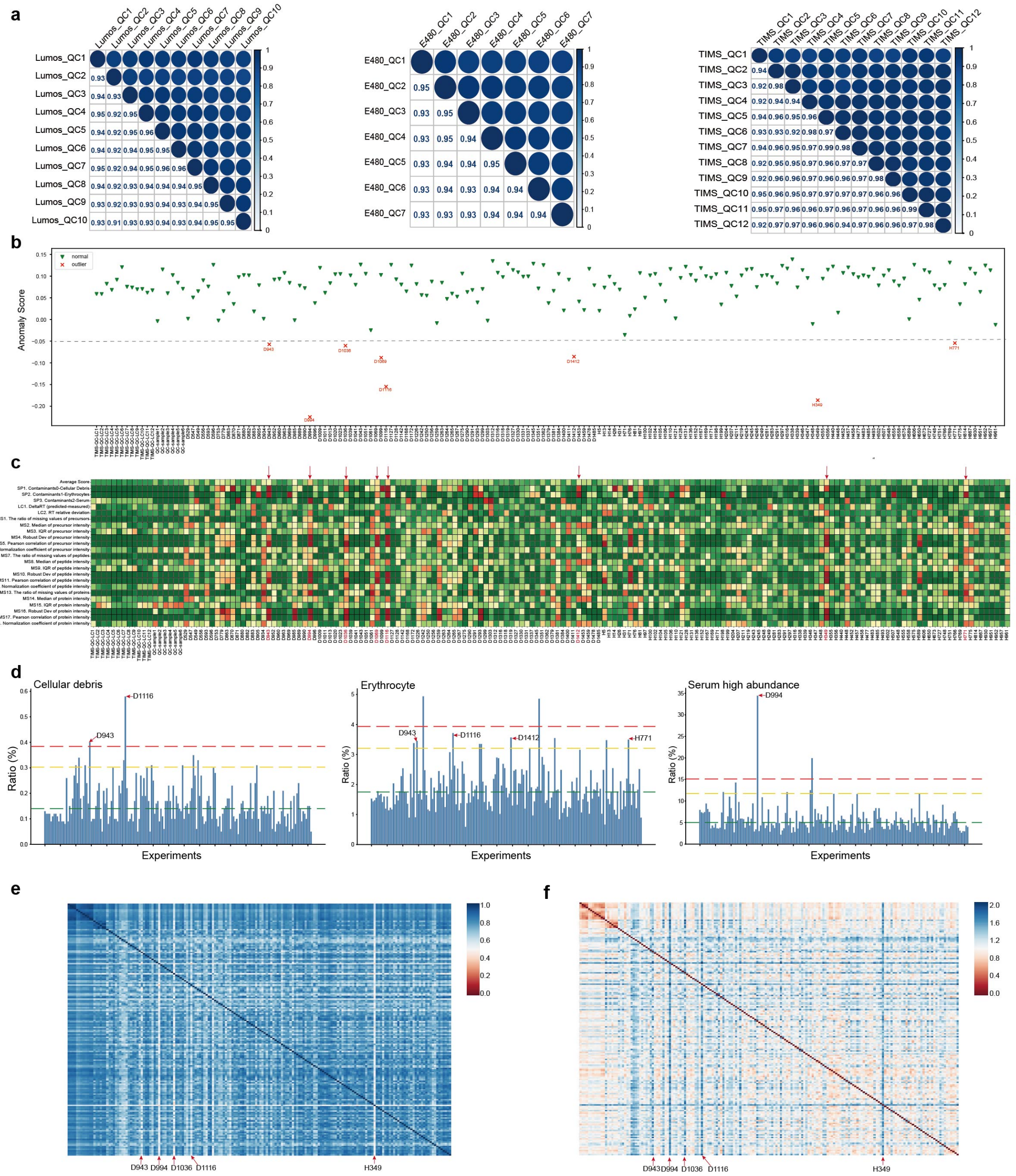

Supplementary Figure 10. Clinical colorectal cancer urinary proteomics analysis in three LC-MS platforms.

(a) Distribution of the number of identified proteins in three LC-MS platforms. QC, quality control samples group; HC, healthy control samples group; CRC, colorectal cancer sample group. **(b)** The unsupervised learning t-Distributed Stochastic Neighbor Embedding (t-SNE) plot overview of urinary proteomics among Lumos, E480, and TIMS, respectively. **(c)** The Orthogonal Projections to Latent Structures Discriminant Analysis (OPLS-DA) plot overview of urinary proteomics among Lumos, E480, and TIMS, respectively. **(d)** Response permutation testing of the model predicted by OPLS-DA among Lumos, E480, and TIMS, respectively. R2 and Q2: parameters of response sequencing test, used to measure whether the model is over fitted. The cross-validation with 100 permutation tests indicated that this OPLS-DA model was reliable. **(e)** The OPLS-DA plot overview of urinary proteomics from three LC-MS platforms together. **(f)** Response permutation testing of the model predicted by OPLS-DA for urinary proteomics data from three LC-MS platforms together.

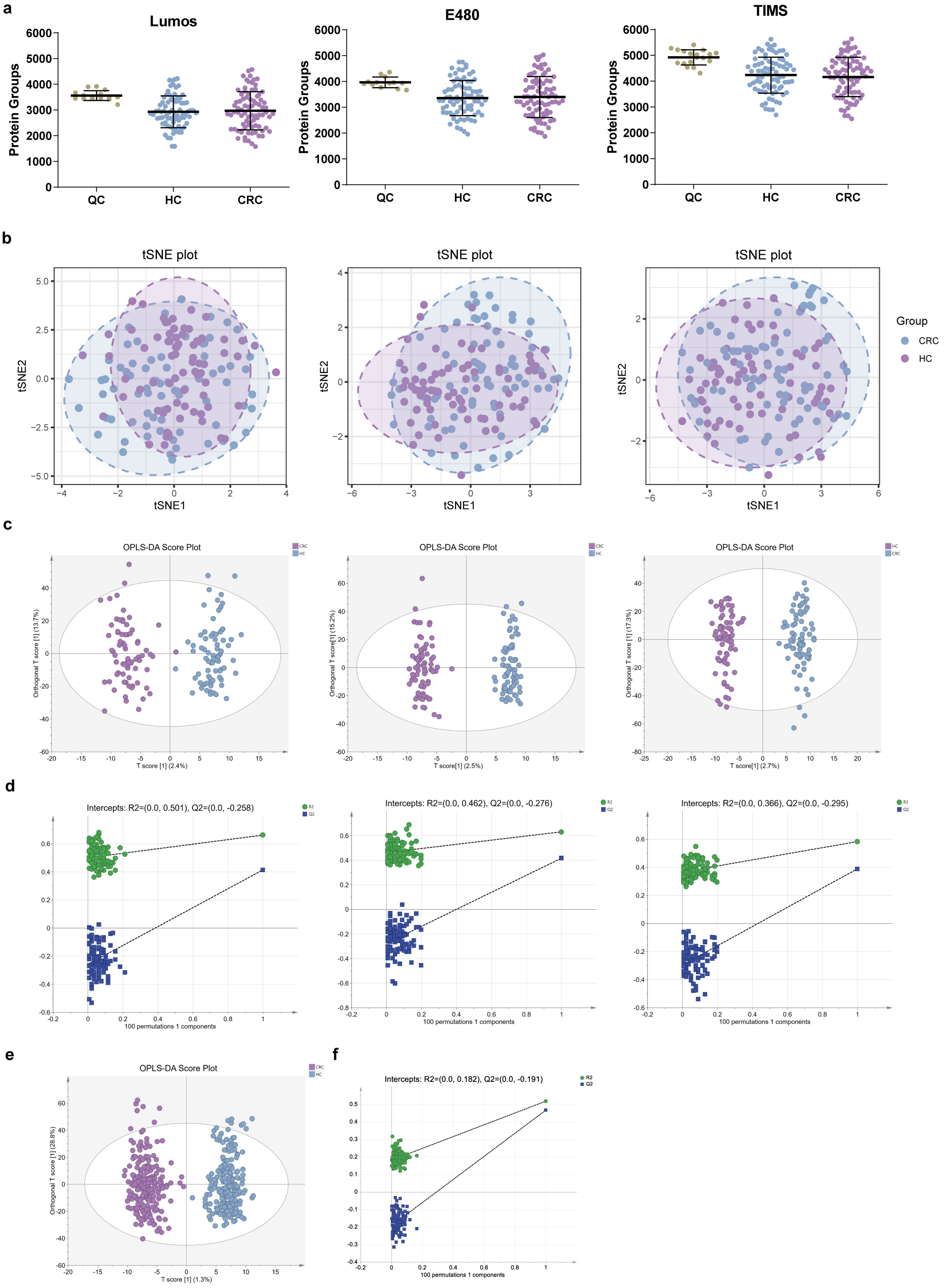

Supplementary Figure 11. GO enrichment analysis and KEGG pathway analysis of urine DEPs between CRC and HC from 3 LC-MS platform and tissue DEPs between CRC and HC from previous study.

(a-c) GO analysis showed the top 15 biological processes (BP, **a**), cellular component (CC, **b**), and molecular function (MF, **c**) sorted by using the BH adjusted *p*-value. **(d)** KEGG analysis showed the top 15 pathways sorted by BH adjusted *p*-value.

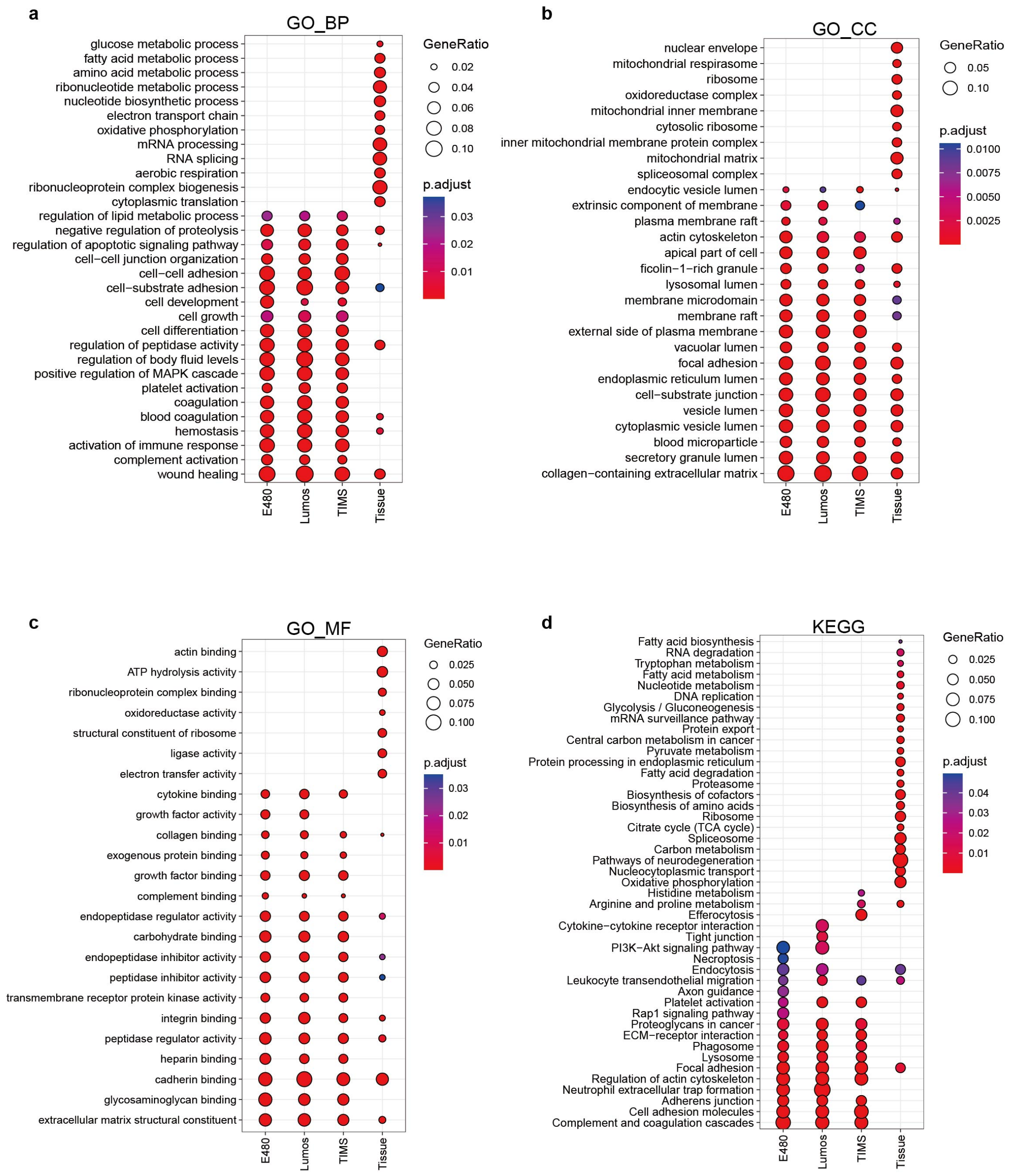

Supplementary Figure 12. Protein-protein interaction network associated with colorectal cancer (CRC).

(a) In the network, the 215 DEPs were classified into 9 groups on the basis of their major functions, including immune system process, complement activation, cell adhesion, metabolic process, signal transduction, cell development, cell differentiation, cell migration, and apoptotic process. The red edge represented the up-regulated protein, and the blue edge represented the down-regulated one. **(b)** The complement activation pathway was significantly activated in CRC. The complement activation includes the classical, lectin, and alternative pathways. Each pathway was activated and amplified by unique stimuli, but they eventually converge when complement protein C3 is cleaved into its C3a and C3b split products, leading to formation of the C5 convertase, production of C5 split products C5a and C5b, and terminal formation of the membrane attack complex (MAC). C3a and C5a are potent signaling molecules, which through their G protein-coupled receptors C3aR and C5aR, respectively, can promote inflammation. MAC, C3a, C5a, and C5aR also promote cell proliferation, differentiation, and inhibit apoptosis.

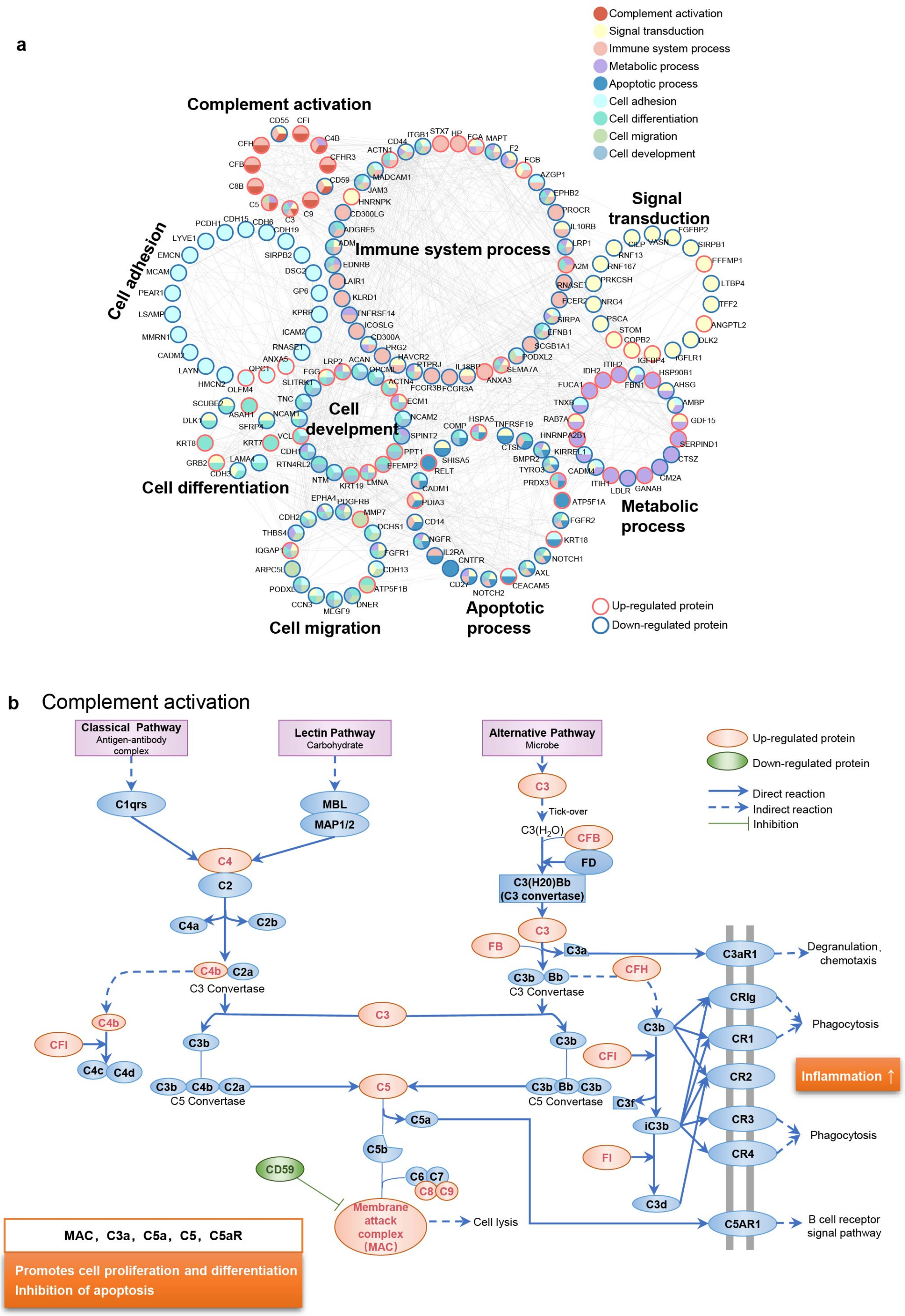

Supplementary Figure 13. Generation of the CRC urinary protein biomarker signature.

(a) The area under the curve (AUC) was used to evaluate the ability of individual proteins to distinguish between CRC patients and healthy control (HC). The top 15 proteins with the highest AUC of individual protein in 3 LC-MS platforms were chosen as candidates. **(b)** The F1 score of six machine learning classifiers trained on one platform and test on the other two platforms, respectively. **(c)** The AUC of six machine learning classifiers trained on one platform and test on the other two platforms, respectively.

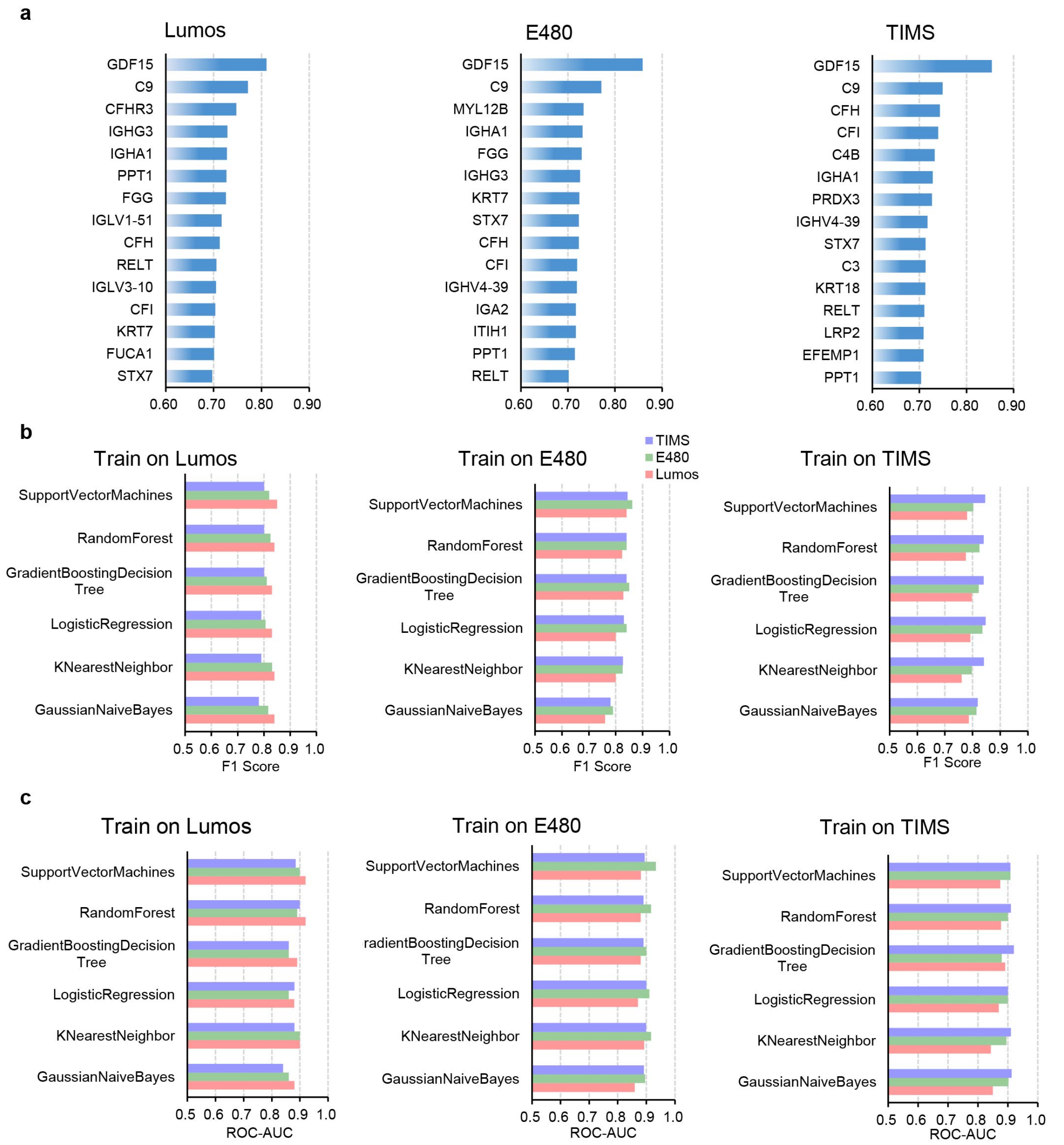

Supplementary Figure 14. The retention time and peak width of pure iRT peptides from 20 LC-MS platforms.

(**a**) The variation of retention times in absolute terms across 10 platforms without LC-SOP is visualized using the 11 iRT peptides. (**b**) The variation of retention times in absolute terms across 10 platforms with LC-SOP is visualized using the 11 iRT peptides. (**c**) The peak width of 11 iRT peptides in 10 platforms without LC-SOP. (**d**) The peak width of 11 iRT peptides in 10 platforms with LC-SOP. Boxplots with the center line, box bounds, and whiskers represent the median, 1st/3rd quartile, and 10th/90th percentile, respectively.

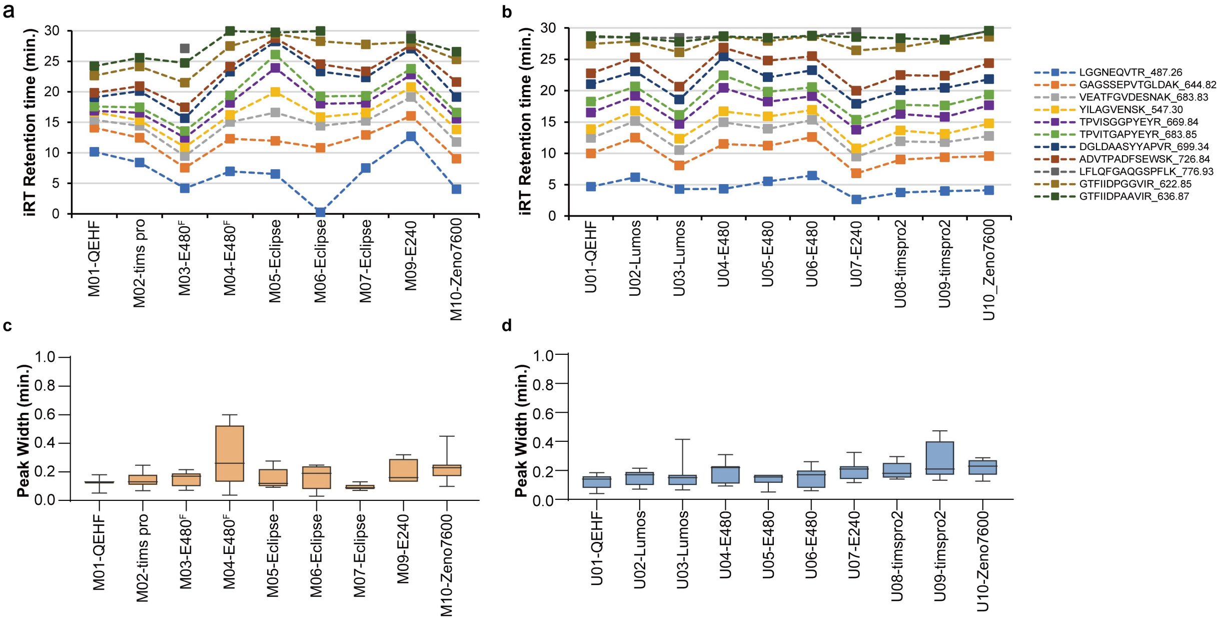

Supplementary Figure 15. The qualitative and quantitative performance of DDA data across 20 LC-MS platforms.

(a) The number of identified proteins (with a 1% FDR) from the 10 platforms without LC-SOP reported by Proteome Discoverer (version 2.4). **(b)** The number of identified proteins (with a 1% FDR) from the 10 platforms with LC-SOP reported by Proteome Discoverer (version 2.4). **(c)** and **(d)** The distribution of the coefficients of variation (CV) obtained on the quantified protein intensity reported by MaxQuant (version 2.2.0.0) across the three technical replicates was plotted for each platform. Boxplots with the center line, box bounds, and whiskers represent the median, 1st/3rd quartile, and 10th/90th percentile, respectively. Horizontal dashed lines were added at 20% CV, as a reference value. **(e)** and **(f)** The Pearson correlation matrix based on log2 quantified protein intensity from 10 platforms without SOP (e) and with SOP (f). The color-scale indicates the magnitude of the Pearson correlation coefficient.

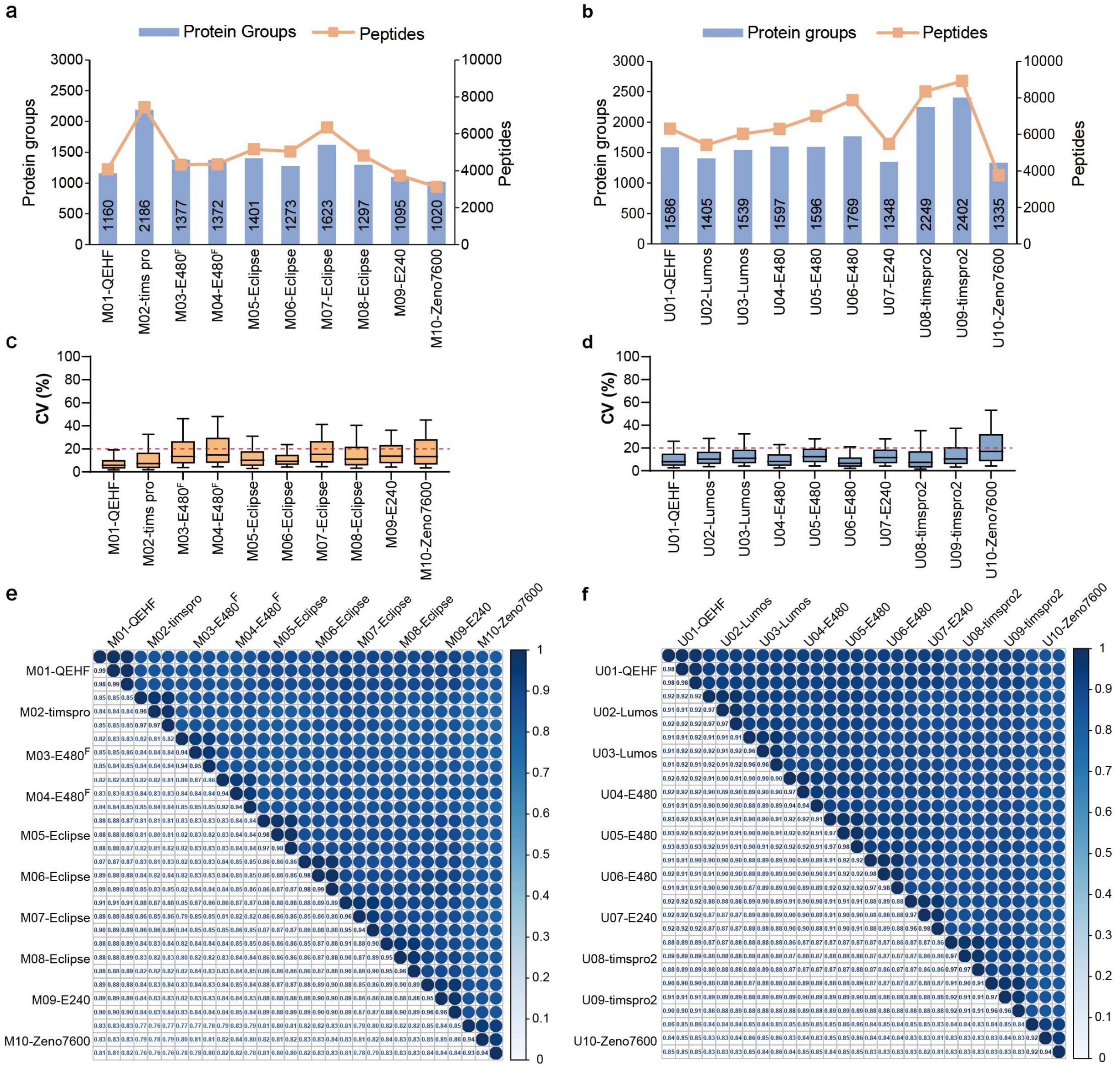

Supplementary Figure 16. The quality assessment of carry-over for LC system for 20 LC-MS platforms.

(a) The ratio of carry-over observed in 10 platforms without LC-SOP. **(b)** The ratio of carry-over observed in 10 platforms with LC-SOP. The ratio of carry-over is calculated by dividing the total protein intensity quantified in blank by the total protein intensity quantified in front DDA run. Generally, low levels of carry-over are observed for 20 LC-MS platforms (<1.5%).

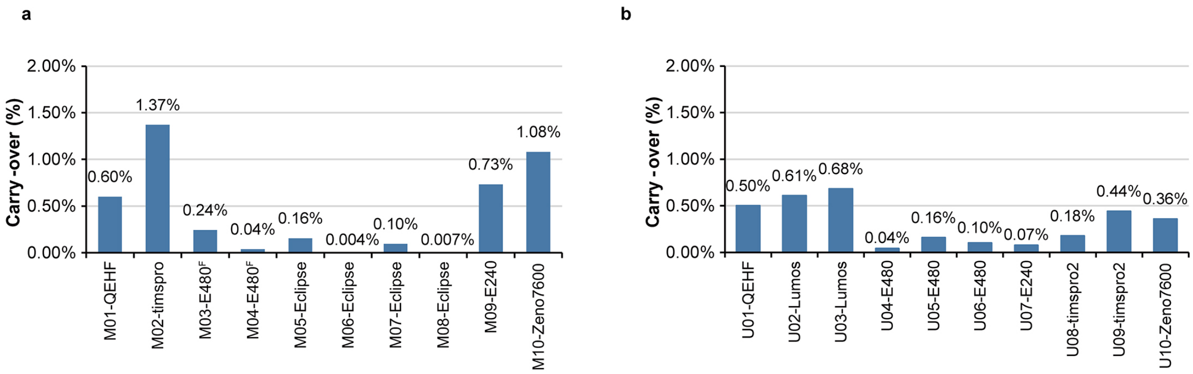

Supplementary Note 1: Comparison of the Metrics in MSCohort and the Existing Metrics in other Quality Control Tools

The NIST MSQC[1] is the most classic quality control (QC) metric for proteomics mass spectrometry experiments, upon which a series of subsequent QC software tools have been developed with additional features and improvements, such as QuaMeter[2, 3], RawBeans[4], DO-MS[5, 6], PTXQC[7], QCloud[8, 9], QC-ART[10], MSstatsQC 2.0[11], and QuiC[12], etc. Additionally, we have developed MSRefine[13], a QC software specifically tailored for Data-Dependent Acquisition (DDA) experiments.

We strive to provide the most comprehensive set of proteomics QC metrics. Our MSCohort software summarizes and integrates metrics extracted by existing QC software and introduces 26 new ones, totaling 81 QC metrics. In **Table S.Note1**, we have compiled a comparison between the 81 QC metrics extracted by MSCohort and existing metrics. In this paper, based on whether the QC metrics are extracted from a single experiment or multiple experiments, all these metrics are classified into two categories: 58 intra-experiment metrics and 23 inter-experiment metrics. Detailed explanations and extraction processes for these 81 metrics are provided in **Supplementary Data 1A and 1B**.

(1) 58 intra-experiment metrics. Existing 48 metrics reported by other tools mainly focus on DDA experiments, few metrics were developed specifically for data-independent acquisition (DIA) experiments. In this study, we proposed 10 new intra-experiment metrics for DIA individual experiments.

We added 3 new metrics related to the DIA acquisition process, including W3. Redundant identified precursors/Identified scan rate, W4. Redundant identified precursors/Identified precursors rate, W5. Identified precursors/Identified scan rate. These metrics are derived from the principles of DIA acquisition and the DIA scoring formula proposed in this study, representing spectra complexity, precursors duplicate identification rate, and the utilization rate of the MS2 scans, respectively. These three metrics directly reflect the quality of the collected DIA data from a data interpretation perspective, playing a crucial role in the quality assessment and optimization of DIA experiments. A detailed explanation of these three metrics can be found in the **Methods** section.

We added 7 new metrics related to the DIA identification results, including S2. Median peptide length, W2. Median of window size (Da), W6. MS1 data points per peak, W7. MS2 data points per peak, M20. Maximum identification rate over m/z range, ID6. Average peptides per protein group, ID7. Average precursors per protein group. These metrics are extracted from the DIA search engine and reflect the quality of the identification results.

In addition to the 10 new metrics mentioned above, the remaining 48 metrics are integrated from existing quality control tools. The quality control software tool extracts relevant metrics based on specific data types (such as DDA or DIA).

According to steps in the LC-MS workflow, the 58 intra-experiment metrics can be assigned to seven categories: sample, chromatography, ion source, DIA windows, dynamic sampling, MS1 and MS2 signal, and identification result. Among them, the metrics in the DIA windows category are applicable only to data-independent acquisition (DIA) experiments, and the metrics in the dynamic sampling category are applicable only to data-dependent acquisition (DDA) experiments, the others are applicable to both DDA and DIA experiments.

(2) 23 inter-experiment metrics. Previous quality control metrics did not specifically consider the contaminants for specific sample type, and did not systematically analyze the variation in

precursors, peptides, and proteins levels. In this study, we added 16 new inter-experiment metrics for comprehensive inter-experiment analysis.

We added customizable contaminants metrics to assess the quality of samples. MSCohort offers configurable lists of custom protein contaminants. In this study, we added 3 contaminants metrics, including erythrocytes[14], cellular debris[15], and serum proteins[16], for monitoring the quality and level of contaminants in urine samples. These 3 contaminants were previously reported as urine-specific quality marker panels to assess the degree of contamination of the urine samples[15]. A detailed explanation of these three sample contaminants metrics can be found in the Methods section. Users can customize and edit the contaminants list based on the actual sample situation. Detailed instructions for making these modifications are provided in the user manual of MSCohort (https://github.com/BUAA-LiuLab/MSCohort).

We added 13 new metrics related to the quantification results in precursors, peptides and proteins level, including the ratio of missing values of precursors/proteins, median of precursors/proteins intensity, IQR (Inter Quartile Range) of precursors/proteins intensity, Pearson correlation of precursors/proteins intensity, robust dev (robust standard deviation)[17] of precursors/ peptides /proteins intensity, and normalization factor of precursors/ peptides /proteins intensity, to comprehensively analyze the consistency and variation of quantification results.

In addition to the 16 new metrics mentioned above, the remaining 7 metrics are integrated from existing quality control tools. These 23 inter-experiment metrics are utilized to assess the stability of chromatography and mass spectrometry across experiments and to detect outlier data. These metrics are universal and can be employed for inter-experiment quality evaluation of both DIA and DDA data, as well as for inter- experiment quality evaluation of Parallel Reaction Monitoring (PRM) data.

Altogether, by extracting the compressive metrics, MSCohort software supports the analysis of data from different types of instrument platforms, including Thermo Scientific Orbitrap, Bruker timsTOF, and SCIEX ZenoTOF. The 81 metrics reported by MSCohort include identification-free (ID-free) metrics directly extracted from .raw/.d/.wiff files, as well as ID-based metrics extracted from identification/quantification results. MSCohort can apply not only to process evaluation and optimization of individual experiments for DDA and DIA data, but also to process quantitative assessment of system performance across multiple experiments for DIA, DDA, and PRM data.

Table S.Note1. List of quality control metrics extracted by MSCohort and compared with other quality control tools. The metrics highlighted in blue font are newly added metrics in MSCohort. According to steps in the LC-MS workflow, the 58 intra-experiment metrics can be assigned to seven categories: sample, chromatography, ion source, DIA windows, dynamic sampling, MS1 and MS2 signal, and identification result. Among them, the metrics in the DIA windows category are applicable only to data-independent acquisition (DIA) experiments, and the metrics in the dynamic sampling category are applicable only to data-dependent acquisition (DDA) experiments, the others are applicable to both DDA and DIA experiments.

**Category**	**58 intra-experiment Metrics**	**MSQC[1]**	**QuaMeter[2, 3]**	**RawBeans[4]**	**DO-MS[5, 6]**	**MSRefine[13]**	**PTXQC[7]**	**QCloud[8, 9]**	**QC-ART[10]**	**QuiC[12]**
**Sample**	S1. Missed cleavages (n=0 of peptides)				√	√	√	√		
	S2. Median peptide length									
	S3. Median total ion chromatography (TIC)	√	√	√		√		√	√	√
	S4. Specificity of digestion					√				
	S5. Frequency of cysteine modification					√				
	S6. Semi/full tryptic peptides	√				√				
	S7. Frequency of contaminants					√	√			
**Chromatography**	C1. Chromatographic invalid acquiring time	√	√			√			√	
	C2. Median of full width at half maximum (FWHM)	√	√	√		√		√	√	√
	C3. Median of peak width	√	√	√		√	√		√	
	C4. Proportion of precursors with long eluting width					√				
	C5. Median precursors identified over RT	√	√		√	√	√		√	
	C6. Maximum identification rate over RT	√	√			√			√	
**DIA Windows (for DIA)**	W1. Acquired MS2 scans in one cycle					√	√			
	W2. Median of window size (Da)									
	W3. Redundant identified precursors/ Identified scan rate									
	W4. Redundant identified precursors/ Identified precursors rate									
	W5. Identified precursors/ identified scan rate									
	W6. MS1 data point per peak									
	W7. MS2 data point per peak									

**Ion Source**	IS1. 1+/2+ ions detected	√	√	√	√	√			√	
	IS2. 3+/2+ ions detected	√	√	√	√	√			√	
	IS3. 4+/2+ ions detected	√	√	√	√	√			√	
	IS4. Median precursor m/z	√	√			√			√	
**Dynamic Sampling (for DDA)**	D1. Scan/peptide rate					√				
	D2. Oversampling once/twice	√	√			√	√			
	D3. Oversampling twice/thrice	√	√			√				
	D4. Maximum redundant scans of one peptide					√				
	D5. MS1 max/sampled for IDs	√	√			√				
**MS1 and MS2 Signal**	M1. Proportion of MS1 time					√				
	M2. Median MS1 ion injection time	√	√	√	√	√		√	√	
	M3. Median MS2 ion injection time	√	√	√	√	√	√	√	√	
	M4. MS1 cycle time		√			√				
	M5. MS2 cycle time		√			√				
	M6. Median peaks intensity of MS1	√	√			√			√	√
	M7. Median peak counts of MS1		√			√				
	M8. Median peak counts of MS2	√	√			√			√	
	M9. Median peaks intensity of MS2					√				√
	M10. Identified / detected features					√		√		
	M11. Signal to noise (S/N for precursors)	√	√						√	
	M12. Median of MS1 raw mass accuracy	√	√	√		√	√	√	√	√
	M13. Median of MS2 raw mass accuracy					√	√			√
	M14. Median intensities for precursors	√	√	√	√	√		√	√	
	M15. Median intensities for peptides					√	√			
	M16. Median intensities for protein groups					√	√			
	M17. Acquired MS1 scans	√	√			√		√	√	
	M18. Acquired MS2 scans	√	√	√		√		√	√	

	M19. MS2 identification rate	√	√			√	√	√	√	
	M20. Maximum identification rate over M/Z range									
	M21. Mean number of tags					√				
	M22. Median PIF				√	√				
**Identification Result**	ID1. Number of Identified MS2 scans	√	√			√			√	
	ID2: Number of precursors	√	√		√	√		√	√	√
	ID3: Number of peptides	√	√			√		√	√	
	ID4. Number of protein groups					√	√	√		√
	ID5. Number of proteins				√	√	√	√		
	ID6. AVG peptides per protein group									
	ID7. AVG precursors per protein group									
**Category**	**23 Inter-experiment metrics**	**MSQC**	**QuaMeter**	**RawBeans**	**DO-MS**	**MSRefine**	**PTXQC**	**QCloud**	**QC-ART**	**QuiC**
**Sample**	SP1. Contaminants——erythrocytes (user-defined)						**√(user-defined)**			
	SP2. Contaminants——cellular debris (user-defined)									
	SP3. Contaminants——serum (user-defined)									
**Chromatography**	LC1. DeltaRT (predicted-measured)									√
	LC2. RT relative deviation						√	√		
**Precursor-level Results**	MS1. The ratio of missing values of precursors									
	MS2. Median of precursor intensity									
	MS3. IQR of precursor intensity									
	MS4. Robust Dev of precursor intensity									
	MS5. Pearson correlation of precursor intensity									
	MS6. Normalization factor of precursor intensity									
**Peptide-level Results**	MS7. The ratio of missing values of peptides								√	
	MS8. Median of peptide intensity						√	√	√	
	MS9. IQR of peptide intensity								√	
	MS10. Robust Dev of peptide intensity									
	MS11. Pearson correlation of peptide intensity								√	

	MS12. Normalization factor of peptide intensity									
**Protein group-level Results**	MS13. The ratio of missing values of proteins									
	MS14. Median of protein intensity						√			
	MS15. IQR of protein intensity									
	MS16. Robust Dev of protein intensity									
	MS17. Pearson correlation of protein intensity									
	MS18. Normalization factor of protein intensity									

Supplementary Note 2: Comparison of MSCohort and other Quality Control Tools

Referring to the review by Wout et al.[18], we divide the currently published quality control tools into two categories: tools evaluating individual experiments and tools comparing multiple experiments.

The first category of tools includes NIST MSQC[1], QuaMeter[2, 3], RawBeans[4], DO-MS[5, 6], which focus on the evaluation and optimization of individual experiments. These tools only extract a limited number of metrics and display them in the form of charts, without quantifying or scoring the metrics. Users need to analyze and understand them on their own. Furthermore, none of these tools propose systematic and comprehensive metrics for deeper proteome profiling and illustrate the relationship between the metrics and identification results. These tools are unable to directly assist users in identifying issues and indicating optimization directions. Users must rely on expert experience to gradually optimize parameters and compare results, a time-consuming and labor-intensive process heavily influenced by human factors, thereby making it challenging to ensure experimental outcomes.

In particular, the above-mentioned software tools primarily focus on the quality evaluation of DDA data, with fewer software options available for DIA data quality control, offering limited metrics and simplistic functionalities.

The second category of tools includes PTXQC[7], QCloud[8, 9], QC-ART[10], MSstatsQC 2.0[11], QuiC[12], which focus on assessing the system performance and variation among multiple experiments. PTXQC and MSstatsQC 2.0 only support the extraction of limited inter-experimental metrics from the identification and quantitative results derived from search engines, and visualize them through charts. QC-ART calculates and reports a distance score using a robust principal components analysis (rPCA) model based solely on user-provided identification and quantification result tables to detect outlier data, lacking detailed reporting and comprehensive inter-experiment variation and quality analysis for each metric. QCloud and QuiC only support specific QC samples or require the inclusion of iRT peptides, performing consistency and bias analysis across experiments solely on peptides specified by the software, rendering them unsuitable for all sample types. These tools have limited scalability and are incapable of conducting comprehensive and systematic analysis for complex large-scale proteomic datasets.

These motivate us to develop MSCohort for comprehensive quality control for proteomics. **Table S.Note2** presents a comparison of MSCohort with existing quality control software tools.

First, we strive to provide the most comprehensive set of proteomics QC metrics. MSCohort software summarizes and integrates metrics extracted by existing QC software and introduces new ones, totaling 81 QC metrics (see Supplementary Note 1 for details).

Second, MSCohort integrates MSRefine[13], a quality control analysis system previously developed by our group for data-dependent acquisition (DDA) single-shot experiment, and establishes a quality control analysis system for DIA single-shot experiment. Consequently, regardless of whether the data is DDA or DIA, MSCohort extracts comprehensive metrics that map to the entire liquid chromatography-mass spectrometry (LC-MS) workflow, illustrates the relationship between extracted metrics and identification results, scores the metrics, and reports visual results, assisting users in evaluating the workflow, and locating problems.

Third, for the cohort proteomics data, MSCohort extracted 23 comprehensive inter-experiment metrics to evaluate multiply experiments. MSCohort also incorporates unsupervised machine learning algorithm (isolation forest) to detect potential outlier experiments. Furthermore, to guarantee the reliability of the subsequent statistical analyses, it incorporates various normalization

methods to remove systematic bias in peptide/protein abundances that could mask true biological discoveries or give rise to false conclusions[19].

Fourth, MSCohort is a powerful tool that supports not only whole LC-MS workflow evaluation and optimization for individual experiments but also robustness assessment and outlier data detecting among multiple experiments. MSCohort also supports comprehensive quality control for different sample types (e.g. cell, tissue, plasma, urine, etc.), different instrument types (Thermo Scientific Orbitrap, Bruker timsTOF, and SCIEX ZenoTOF), and different acquisition modes (DDA, DIA, PRM).

Table S.Note2. An overview of the computational quality control tools for LC-MS based proteomics

Tool	Number of metrics	Experiment type	Instrument	ID-free metrics	ID-based metrics	Main Functions	Detailed Functions
**MSQC**[1]	46	DDA	Thermo	√	√	Tools evaluating individual experiments	• Extract metrics
**QuaMeter**[2, 3]	45	DDA	Thermo, Bruker, SCIEX	√	√	Tools evaluating individual experiments	• Extract metrics
**RawBeans**[4]	13	DDA, DIA	Thermo, Bruker, SCIEX	√	√	Tools evaluating individual experiments	• Extract metrics<br>• Generate visual report
**DO-MS**[5, 6]	35	DDA, DIA	Thermo, Bruker	√	√	Tools evaluating individual experiments	• Extract metrics<br>• Generate visual report
**MSRefine**[13]	47	DDA	Thermo, Bruker, SCIEX	√	√	Tools evaluating individual experiments	• Extract metrics<br>• Provide a scoring system<br>• Illustrate the relationship between metrics and identification results<br>• Generate visual report
**PTXQC**[7]	24	DDA	Thermo, Bruker, SCIEX		√	Tools comparing multiple experiments	• Extract metrics<br>• Compute the quality score<br>• Generate visual report
**QCloud**[8, 9]	23	DDA, PRM, SRM	Thermo, Bruker, SCIEX		√	Tools comparing multiple experiments	• Extract metrics<br>• Generate visual report
**QC-ART**[10]	User-input	DDA	Not defined		√	Tools comparing multiple experiments	• Detect outlier experiments<br>• Generate visual report
**MSstatsQC 2.0**[11]	User-input	DDA, DIA, SRM	Not defined		√	Tools comparing multiple experiments	• Generate visual report
**QuiC**[12]	22	DDA, DIA, MRM, PRM	Thermo, Bruker, SCIEX	√	√	Tools comparing multiple experiments	• Extract metrics<br>• Generate visual report
**MSCohort (This study)**	81	DDA, DIA, PRM	Thermo, Bruker, SCIEX	√	√	**Tools evaluating individual experiments and comparing multiple experiments**	• Extract metrics<br>• Provide a scoring system<br>• Illustrate the relationship between metrics and identification results<br>• Compute the quality score<br>• Generate visual report<br>• Detect outlier experiments<br>• Data normalization

Supplementary Note 3: The Standard Operating Procedure (SOP) for Urinary Proteomics

S.Note 3.1: Sample Preparation of the Urine Samples

First-morning urine (midstream) samples were collected from each participant. All of the samples were centrifuged at 3000 × g for 30 minutes at 4 °C to remove cell debris. The supernatant was transferred into the 2 mL EP tube (Corning, USA) and stored at −80 °C for further analysis.

Urine peptides were prepared by the 96 DRA-Urine (direct reduction/alkylation in urine with 96-well PVDF plate) method, as reported in our previous work[20]. For each sample, two milliliters of urine were reduced with 20 mM dithiothreitol (DTT) for 5 min at 95 °C, and then alkylated with 50 mM iodoacetamide (IAM) at room temperature (RT) in the dark for 45 min. Subsequently, 12 mL precooled acetone was added to each sample and placed at -20 °C for 30 min to extract the urine proteins. Then, all of the samples were centrifuged at 10,000 × g for 10 min at 4 °C. The protein precipitate was re-dissolved in 200 μL of 20 mM Tris. The concentration of urine proteins was quantified using Pierce™ BCA protein assay kit (Thermo Fisher Scientific, USA) following the manufacturer's protocol. During protein precipitation and quantification, each well of the 96-well PVDF plate (MSIPS4510, Millipore, Billerica, MA) was prewetted with 150 μL of 70% ethanol and equilibrated with 300 μL of 20 mM Tris. For each well, one hundred micrograms of proteins were transferred to the 96-well PVDF plate. The protein samples were then washed three times with 200 μL of 20 mM Tris buffer (pH 8.0) and centrifuged at 3000 rpm for 5 min (Hunan Kecheng Instrument Equipment Co., Ltd). For per well, proteins were digested by adding 30 μL of 20 mM Tris buffer (pH 8.0) containing 2 μg trypsin on the membrane. The samples were subjected to microwave-assisted protein enzymatic digestion twice in a water bath for 1 min under microwave irradiation[21] and then at 37 °C water bath for 2 h. Subsequently, the resulting peptides were collected by centrifugation at 3000 rpm for 5 min. Then eluted peptides were quantified using Pierce™ Quantitative Colorimetric Peptide Assay kit (Thermo Scientific) following the manufacturer's protocol. Peptides were aliquoted into 20 μg/tube and lyophilized and stored at −80 °C for further LC-MS analysis.

Before LC-MS analysis, the peptides were resuspended in 20 μL of 0.1% FA in H_2O. Eleven non-naturally occurring synthetic peptides from the iRT kit (Biognosys) were spiked into each sample at a ratio of 1:30 (v/v) to correct relative retention times between acquisitions. The urine quality control (QC) sample was generated by pooling 5 μL of each sample to monitor the reproducibility of the LC-MS system. Finally, 2 μg peptide was subjected to LC-MS analysis.

S.Note 3.2: Preparation for System Suitability Tests

In order to standardize the acquisition protocol and to generate an initial quality assessment, all LC-MS platforms were asked to perform two different system suitability tests prior to starting the main study. Three different sets of samples needed to be prepared before data acquisition, 1) a pure iRT sample containing 11 non-naturally occurring synthetic peptides (Biognosys) to assess nanoflow LC system suitability, 2) a predigested urine peptide QC sample to assess general system performance, 3) blank solution to assess carry-over of the LC system.

iRT mixture (stock solution) (based on supplier instructions)

- Add 50 μL dissolution buffer (blue cap) to the iRT standard tube (red cap).
- Vortex the iRT standard tube for at least 1 minute.
- Subject tube to 5 min ultrasonic bath.
- Store iRT mixture (stock solution) at 2-8°C (stable for 12 weeks).

- Prepare pure iRT solution by taking 1 µL of the stored iRT mixture and adding 29 µL H_2O (+0.1% FA).
- Load 2 µL of pure iRT peptides to LC-MS analysis.

Predigested urine QC peptide stock solution (1 µg/µL - 20 µL)
- Defrost urine QC peptide (containing iRT) at room temperature for 15 min.
- Centrifugate the tube at 10000 × g for 5 min.
- Add 20 µL H_2O (+0.1% FA) to the tube.
- Vortex the tube for at least 30 s.
- Load 2 µL of urine peptides to LC-MS analysis for each experiment.

Blank solution
- Add 100 µL H_2O (+0.1% formic acid) to a LC vial.
- Put LC vial in autosampler. Load 2 µL of blank solution to LC-MS analysis.

S.Note 3.3: iRT Nanoflow LC System Suitability Test

First, the pure iRT test was performed to evaluate the LC separation and the sensitivity and mass accuracy of MS data acquisition, and ensure the nanoflow LC and MS system are working well.

3.3.1 LC Method

- Solvent A: 0.1 % FA in Water.
- Solvent B: 0.1 % FA in 80 % Acetonitrile.
- Wash solvent: 0.1 % FA in 80 % Acetonitrile.
- Analytical column: 50cm × 50µm monolithic silica capillary column (Beijing Uritech Biotech)
- Temperature (EASY- SPRAY source): 60°C.
- Temperature autosampler: 4°C.
- Loading amount: 2 µL
- Gradient:

Time (min.)	Flow (nL/min.)	%B
0	500	5
22	500	20
25.3	500	30
25.4	500	90
26.4	500	90
26.5	500	2
30	500	2

3.3.2 MS Method

iRT peptide data acquisition was conducted in DDA mode. The detailed MS parameters were listed for different types of MS (Orbitrap, timsTOF, ZenoTOF).

1) MS parameters for Orbitrap

Category	Parameters	Settings
Global Settings	Acquisition time	30 min
	Acquisition mode	DDA
	Advanced Peak Determination	TRUE

	FAIMS Mode	Not installed
**MS Settings**	Detection type for full MS	Orbitrap
	Resolution for full MS	60000
	Scan range for full MS (m/z)	350 - 1200
	Maximum injection time for full MS	20ms
	AGC target for full MS	custom
	Normalized AGC target for full MS	250%
	Minimum intensity	5000
	cycle time	1s
	Include charge state	2-6
	Exclusion duration	15s
	Data dependent mode	Cycle time
**MS/MS Settings**	Activation type	HCD
	Isolation window(m/z)	1.6
	Normalized collision energy (%)	32
	Detection type for MS2	Orbitrap
	Resolution for MS2	15000
	Fixed first mass for MS2	110
	AGC target for MS2	custom
	Normalized AGC target for MS2	75%
	Maximum injection time for MS2(ms)	22ms

2) MS parameters for timsTOF

Category	Parameters	Settings
**Global Settings**	Acquisition time	30min
	Acquisition mode	DDA
	Detection type for MS	TOF
**MS Settings**	Scan Mode	PASEF
	Scan range for MS (m/z)	100-1700
**TIMS Settings**	1/K0 start (V.s/cm^2)	0.75
	1/K0 End (V.s/cm^2)	1.3
	Ramp time	100ms
	Accu. Time	100ms
	Duty cycle	100%
	Ramp rate	9.43Hz
**MS/MS Settings**	Number of PASEF ramps	9
	Total Cycle time	1.06 s
	Charge minimum	2
	Charge maximum	5
	Target intensity	5000
	Intensity Threshold	1000
**Active Exclusion**	Active exclusion	TRUE
	Release after	0.25min
**Collision Energy Setting**	1/k0 (V.s/cm2)	0.6; 1.6
	Collision energy	20; 59
**Isolation Width Setting**	mass (m/z)	700; 800
	width (m/z)	2; 3

3) MS parameters for ZenoTOF

Category	Parameters	Settings
**Global Settings**	Acquisition time	30 min
	Acquisition mode	DDA
	Detection type for MS	TOF
	Total scan time (seconds)	1.368
	Estimated cycles	1316
**MS Settings**	Scan type	TOFMS
	Polarity	Positive
	Spray voltage (V)	3000
	Scan range for MS	350-1200
	Accumulation time (s)	0.1
	Declustering potential (V)	80
	Collision energy (V)	10
	Time bins to sum	8
**IDA Criteria**	Maximum candidates ion	50
	Intensity threshold exceeds (counts/s)	100
	Exclude former candidate ions	TRUE
	For (sec)	12
	After (occurrences)	1
	Dynamic CE for MS/MS	TRUE
	Charge state	2-5
	Isotope to select	Monoisotopic
**IDA Dependent**	Scan type	TOFMSMS
	Polarity	Positive
	Fragmentation mode	CID
	Scan range for MS/MS	110-1500
	Accumulation time (s)	0.02
	Zeno threshold (cps)	100000
	Declustering potential (V)	80
	Tims bins to sum	8

3.3.3 Data Evaluation

1) Open the data file in Xcalibur (Thermo Scientific), DataAnalysis (Bruker Daltonik), Peakview (SCIEX) and extract the table of ions from the MS data.

Center	Compound	ID
487.257	LGGNEQVTR	iRT Kit_a
644.822	GAGSSEPVTGLDAK	iRT Kit_b
683.827	VEATFGVDESNAK	iRT Kit_c
547.297	YILAGVENSK	iRT Kit_d
669.838	TPVISGGPYEYR	iRT Kit_e
683.853	TPVITGAPYEYR	iRT Kit_f
699.338	DGLDAASYYAPVR	iRT Kit_g
726.835	ADVTPADFSEWSK	iRT Kit_h
622.853	GTFIIDPGGVIR	iRT Kit_i
636.869	GTFIIDPAAVIR	iRT Kit_k
776.929	LFLQFGAQGSPFLK	iRT Kit_l

2) Ensure that the peak shape and widths look good, average peak width ~ 0.12 min at half height. Make sure the retention times look reasonable, good separation of peaks, no big dead

volume causing the peaks to come out very late, minimal peak tailing, etc.

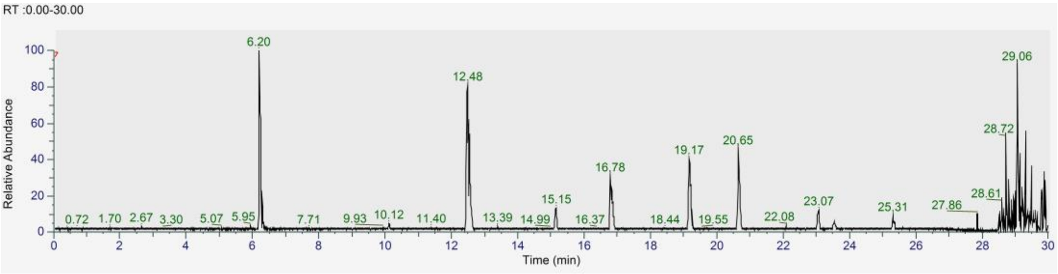

3) Ensure the mass accuracy observed on average for the MS1 peaks at peak apex is better than 5 ppm. (e.g. extract m/z 487.257)

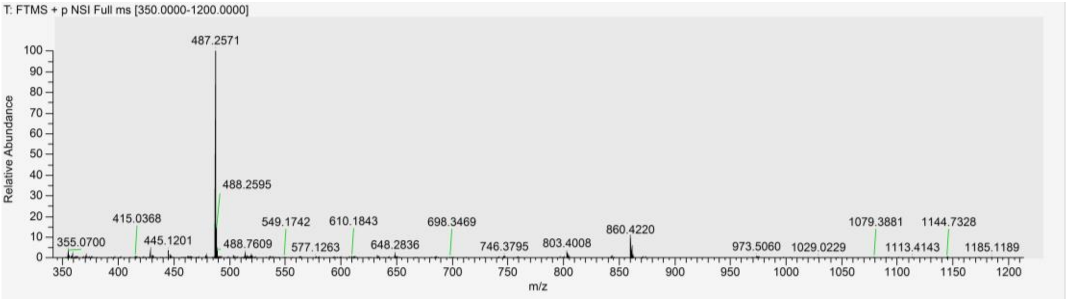

For 10 LC-MS platforms with LC-SOP (U01-U10), the retention times and peak widths of pure iRT peptides from 10 LC-MS platforms were shown in Supplementary Figure 14.

3.3.4 File Naming

For 10 LC-MS platforms with LC-SOP (U01-U10), to facilitate the organization of the data as well as the processing of the data, each platform will be assigned a site number. Please use a file naming convention that includes an acronym that identifies your platform, the sample injected, and the replicate number, etc.

For example, for the iRT sample acquired at your platform #1, the file name contains your platform number and looks as follows: U01_QEHF_iRT.raw

S.Note 3.4: Human Urine QC Sample Performance Test

After performing the iRT test, the urine QC sample test could be performed for comprehensive evaluation of the LC and MS system.

3.4.1 LC Method

The LC method for urine QC sample test is the same as the iRT test.

- Solvent A: 0.1 % FA in Water.
- Solvent B: 0.1 % FA in 80 % Acetonitrile.
- Wash solvent: 0.1 % FA in 80 % Acetonitrile.
- Analytical column: 50cm×50μm monolithic silica capillary column (Beijing Uritech Biotech)
- Temperature (EASY- SPRAY source): 60°C.
- Temperature autosampler: 4°C.
- Loading amount: 2 μL
- Gradient:

Time (min.)	Flow (nL/min.)	%B
0	500	5

22	500	20
25.3	500	30
25.4	500	90
26.4	500	90
26.5	500	2
30	500	2

3.4.2 MS Method

Human urine QC sample data acquisition was conducted in DIA mode. The DIA acquisition method adopted a variable window size consisting of 80 DIA windows for Orbitrap and ZenoTOF data and 50 windows for timsTOF data. In order to establish a data acquisition method suitable for large cohort samples, we adopted an acquisition time of 30 minutes, balancing the high throughput and high identification depth. Ten LC-MS platforms in with LC-SOP group, employed a unified LC condition and consistent MS parameters for the same type of instrument.

1) MS parameters for Orbitrap

Category	Parameters	Settings
**Global Settings**	Acquisition mode	DIA
	Advanced Peak Determination	TRUE
	FAIMS Mode	Not installed
**MS Settings**	Detection type for full MS	Orbitrap
	Resolution for full MS	120000
	Scan range for full MS	350-1200
	Maximum injection time for full MS	50ms
	AGC target for full MS	1000000
	Normalized AGC target for full MS (%)	300%
	cycle time(s)	3s
**MS/MS Settings**	Activation type	HCD
	Isolation window (m/z)	custom
	Normalized collision energy (%)	30
	Detection type for MS2	Orbitrap
	Resolution for MS2	30000
	Fixed first mass for MS2	120
	AGC target for MS2	custom
	Normalized AGC target for MS2(%)	200%
	Maximum injection time for MS2(ms)	50ms
	DIA windows number	80 variable windows

Full inclusion list for DIA scans

m/z	Isolation Window	Polarity
Full MS	[350-1200]	Positive
358.5	17	Positive
373.5	13	Positive
384.5	9	Positive
395	13	Positive
404.5	7	Positive
411.5	7	Positive
419	8	Positive
426	6	Positive

432	6	Positive
437.5	5	Positive
442.5	5	Positive
447.5	5	Positive
452.5	5	Positive
458	6	Positive
463.5	5	Positive
469	6	Positive
474	4	Positive
479	6	Positive
484	4	Positive
488.5	5	Positive
493	4	Positive
498	6	Positive
503	4	Positive
507	4	Positive
511.5	5	Positive
516	4	Positive
Full MS	[350-1200]	Positive
521	6	Positive
526	4	Positive
530.5	5	Positive
536	6	Positive
541	4	Positive
545	4	Positive
549.5	5	Positive
554	4	Positive
558.5	5	Positive
564	6	Positive
570	6	Positive
576	6	Positive
581.5	5	Positive
587	6	Positive
593	6	Positive
598.5	5	Positive
603.5	5	Positive
609	6	Positive
614.5	5	Positive
619.5	5	Positive
625.5	7	Positive
631.5	5	Positive
636.5	5	Positive
641.5	5	Positive
647	6	Positive
653.5	7	Positive
Full MS	[350-1200]	Positive
659.5	5	Positive
665.5	7	Positive
672	6	Positive
678.5	7	Positive
686.5	9	Positive

694.5	7	Positive
702.5	9	Positive
711	8	Positive
719	8	Positive
728	10	Positive
738	10	Positive
748	10	Positive
758.5	11	Positive
768.5	9	Positive
779	12	Positive
791.5	13	Positive
803.5	11	Positive
815	12	Positive
828	14	Positive
843	16	Positive
859.5	17	Positive
876.5	17	Positive
898.5	27	Positive
923.5	23	Positive
943.5	17	Positive
962.5	21	Positive
997.5	49	Positive
1061	78	Positive

2) MS parameters for timsTOF

Category	Parameters	Settings
**DIA Method**	Scan Mode	dia-PASEF
	Scan range for MS	100-1700 m/z
**TIMS Settings**	1/K0 start (V.s/cm^2)	0.75
	1/K0 End (V.s/cm^2)	1.3
	Ramp time	100 ms
	Accu. Time	100 ms
	Duty cycle	100
	Ramp rate	9.43 Hz
**MS/MS Windows Setting**	MS width	16 Da
	MS overlap	0 Da
	Mass steps per cycle	50
	Calculate from polygon	Mass steps
	Mobility overlap	0 1/k0
	1/k0 (V.s/cm2)	0.6; 1.6
	Collision energy	20; 59
	Mobility range (1/k0)	0.75-1.29
	Mass range	369-1169
	Mean cycle time estimate	1.48 s

Full inclusion list for DIA scans

Cycle Id	Start IM [1/K0]	End IM [1/K0]	Start Mass [m/z]	End Mass [m/z]
1	1.2289	1.2849	1105	1121
1	1.0851	1.2199	897	913

1	0.9523	1.0766	705	721
1	0.8305	0.9452	529	545
1	0.7501	0.8258	369	385
2	1.2400	1.2847	1121	1137
2	1.0961	1.2318	913	929
2	0.9633	1.0885	721	737
2	0.8416	0.9571	545	561
2	0.7500	0.8377	385	401
3	1.2511	1.2846	1137	1153
3	1.1072	1.2437	929	945
3	0.9744	1.1004	737	753
3	0.8527	0.9691	561	577
3	0.7500	0.8497	401	417
4	1.2621	1.2844	1153	1169
4	1.1183	1.2557	945	961
4	0.9855	1.1124	753	769
4	0.8637	0.9810	577	593
4	0.7531	0.8616	417	433
5	1.1293	1.2676	961	977
5	0.9965	1.1243	769	785
5	0.8748	0.9930	593	609
5	0.7641	0.8736	433	449
6	1.1404	1.2796	977	993
6	1.0076	1.1363	785	801
6	0.8859	1.0049	609	625
6	0.7752	0.8855	449	465
7	1.1625	1.2859	1009	1025
7	1.0297	1.1601	817	833
7	0.9080	1.0288	641	657
7	0.7863	0.8974	465	481
8	1.1736	1.2857	1025	1041
8	1.0408	1.1721	833	849
8	0.9191	1.0407	657	673
8	0.7973	0.9094	481	497
9	1.2068	1.2852	1073	1089
9	1.0629	1.1960	865	881
9	0.9301	1.0527	673	689
9	0.8084	0.9213	497	513
10	1.2179	1.2851	1089	1105
10	1.0740	1.2079	881	897
10	0.9412	1.0646	689	705
10	0.8195	0.9333	513	529
11	1.1515	1.2860	993	1009
11	1.0187	1.1482	801	817
11	0.8969	1.0169	625	641
12	1.1847	1.2856	1041	1057
12	1.0519	1.1840	849	865
13	1.1957	1.2854	1057	1073

3) MS parameters for ZenoTOF

Category	Parameters	Settings
**DIA Method**	Acquisition time	30 min
	Acquisition mode	SWATH
	Total scan time (seconds)	2.095
	Estimated cycles	859
**MS Settings**	Scan type	TOFMS
	Polarity	positive
	Spray voltage (V)	3000
	Scan range for MS	350-1200
	Accumulation time (s)	0.25
	Declustering potential (DP) (V)	80
	Collision energy (CE) (V)	10
	Time bins to sum	8
**TOF MS/MS**	Fragmentation mode	CID
	Scan range for MS/MS	120-1500
	Accumulation time (s)	0.018
	Dynamic collision energy	TRUE
	Time bins to sum	8
	Scan windows number	80 variable windows

Full inclusion list for DIA scans

m/z	Isolation Window	DP	CE
350-367	17	80	16
367-380	13	80	17
380-389	9	80	18
389-401	2	80	18
401-408	7	80	19
408-415	7	80	19
415-423	7	80	19
423-429	6	80	20
429-435	6	80	20
435-440	5	80	20
440-445	5	80	21
445-450	5	80	21
450-455	5	80	21
455-461	6	80	21
461-466	5	80	22
466-472	6	80	22
472-476	4	80	22
476-482	6	80	22
482-486	4	80	23
486-491	5	80	23
491-495	4	80	23
495-501	6	80	23
501-505	4	80	24
505-509	4	80	24
509-514	5	80	24
514-518	4	80	24
518-524	6	80	24
524-528	4	80	25

528-533	5	80	25
533-539	6	80	25
539-543	4	80	25
543-547	4	80	26
547-552	5	80	26
552-556	4	80	26
556-561	5	80	26
561-567	5	80	27
567-573	6	80	27
573-579	6	80	27
579-584	5	80	27
584-590	6	80	28
590-596	6	80	28
596-601	5	80	28
601-606	5	80	28
606-612	6	80	29
612-617	5	80	29
617-622	5	80	29
622-629	7	80	30
629-634	5	80	30
634-639	5	80	30
639-644	5	80	30
644-650	6	80	31
650-657	7	80	31
657-662	5	80	31
662-669	7	80	32
669-675	6	80	32
675-682	7	80	32
682-691	9	80	32
691-698	7	80	33
698-707	9	80	33
707-715	8	80	34
715-723	8	80	34
723-733	10	80	35
733-743	10	80	35
743-753	10	80	35
753-764	11	80	36
764-773	9	80	37
773-785	12	80	37
785-798	13	80	38
798-809	11	80	38
809-821	12	80	39
821-835	14	80	39
835-851	16	80	40
851-868	17	80	41
868-885	17	80	42
885-912	17	80	43
912-935	13	80	44
935-952	17	80	45
952-973	21	80	46
973-1022	49	80	47
1022-1100	78	80	50

3.4.3 Data Evaluation

1) Data processing of urine proteome with Spectronaut

The urine QC sample test data was processed with Spectronaut v.18.0 performing the directDIA analysis. All searches were performed against the human SwissProt database (Homo sapiens, 20386 reviewed entries, 2022_06 version), concatenated with iRT peptide.fasta file (downloaded from the Biognosys webpage). Default settings were used unless otherwise noted. Cross-run normalization was turned off. Carbamidomethyl on C was set as fixed modification, and Oxidation on M as variable modification.

The data processing results were exported and further analyzed using MSCohort. The customized reports required in MSCohort were provided in the user manual of MSCohort software (https://github.com/BUAA-LiuLab/MSCohort).

2) Data quality evaluation of urine proteome with MSCohort

The urine QC sample test data quality evaluation was processed with MSCohort. The download link and user manual of MSCohort were provided on Github (https://github.com/BUAA-LiuLab/MSCohort).

1. MSCohort download

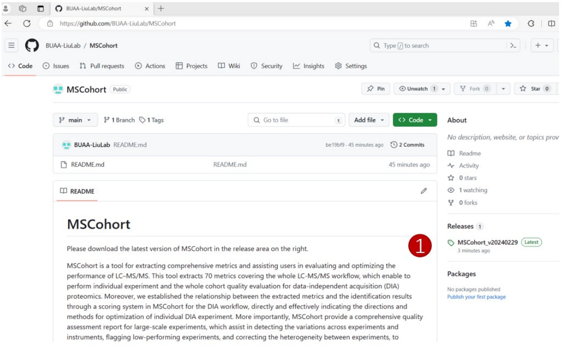

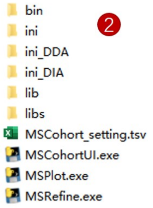

① Login https://github.com/BUAA-LiuLab/MSCohort and download the lasted version at the right of the page.

② The unzipped MSCohort file.

For individual urine QC sample test experiment, all participants performed intra-experiment analysis through MSCohort to evaluate the data quality systematically.

1. Analyzing with MSCohort

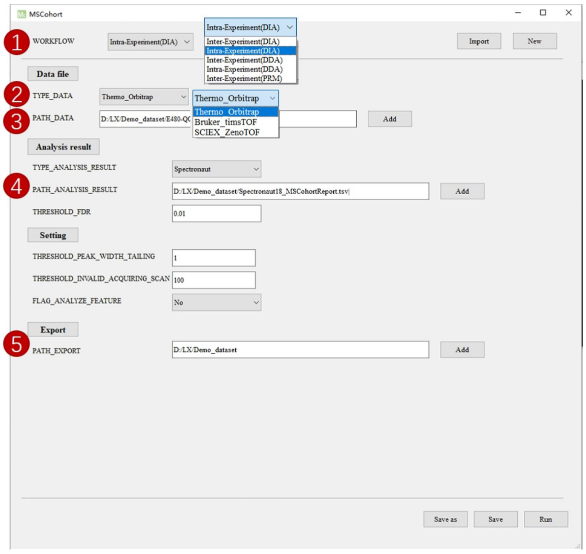

① Select **WORKFLOW** as **Intra-experiment(DIA)**;

② Select **TYPE_DATA** according to the data type ;

③ Click Add to select the raw file into the **PATH_DATA**;

④ Click Add to select the Spectronaut customized report for MSCohort into the **PATH_ANALYSIS_RESULT**;

⑤ Click Add to set the **PATH_EXPORT** for saving the results.

Note:

Space (“ ”) cannot exist in the file directory (including PATH_DATA, PATH_ANALYSIS_RESULT, and PATH_EXPORT), which will affect the normal running of the program.

The MSCohort report and metrics-score diagram directly and effectively indicate the overall performance of the urine QC sample test data. If any metrics showed a poor score (< 2) (The detailed description of the metrics is shown in **Supplementary Data 1**), the data collection and assessment steps will be repeated after fixing specific issues based on the report, until passing the quality control assessment.

2. MSCohort Results

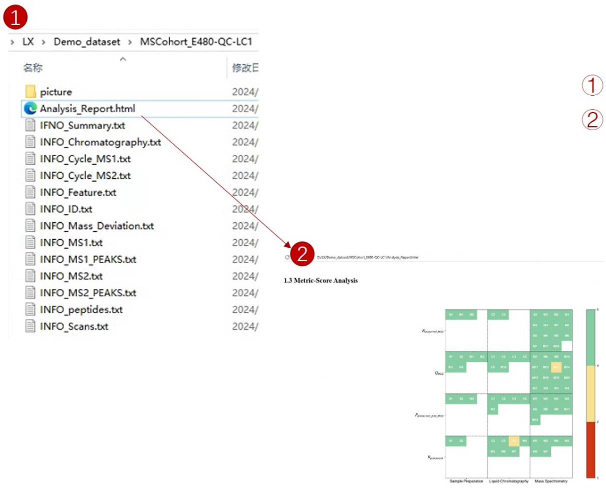

① The MSCohort results ;

② Double clicking **Analysis_Report.html**, the report will be preformed in the browser.

The data arising from the analysis of this initial phase was used to improve the data acquisition and prompt any platforms that have any issues.

S.Note 3.5: Data Acquisition for Actual Samples

3.5.1 Data Acquisition

After passing the quality control assessment, the subsequent actual samples were performed using the same acquisition method as the urine QC sample (see S.Note 1.4: Human Urine QC Sample Performance Test for details).

For 10 LC-MS platforms with LC-SOP (U01-U10). To systematically analyze and compare the reproducibility of DDA and DIA data across multiple platforms, the DDA data was performed in triplicate (Supplementary Figure 5), one blank data was performed to assess carry-over (Supplementary Figure 6), and then the DIA data was performed in triplicate. Finally, the datasets were assembled centrally and processed.

Run order for 10 LC-MS platforms with LC-SOP:

Sample	Gradient	Mode
pure iRT sample	30min	DDA
Urine peptide QC sample	30min	DDA
Urine peptide QC sample	30min	DDA
Urine peptide QC sample	30min	DDA
blank	30min	DDA
Urine peptide QC sample	30min	DIA
Urine peptide QC sample	30min	DIA
Urine peptide QC sample	30min	DIA
blank	30min	DIA

File Naming

For 10 LC-MS platforms with LC-SOP (U01-U10), to facilitate the organization of the data as well as the processing of the data, each platform will be assigned a site number. Please use a file naming convention that includes an acronym that identifies your platform, the sample injected, and the replicate number, etc.

For example, for the urine peptide QC sample acquired at your platform #1, the file name contains your platform number and looks as follows: U01_QEHF_DIA_R1.raw

3.5.2 Data Evaluation

After each sample was collected, the quality of individual experiment data could be assessed using MSCohort intra-experiment analysis (see S.Note 1.4: Human Urine QC Sample Performance Test for details).

Finally, after all the samples data were collected, the datasets were assembled centrally and processed with Spectronaut. The data processing results were exported and further inter-experiment analysis was performed using MSCohort to comprehensively evaluate the stability and reproducibility of data acquisition across multiple experiments, including retention time stability, Pearson correlation, quantitative precision, etc.

2. Analyzing with MSCohort

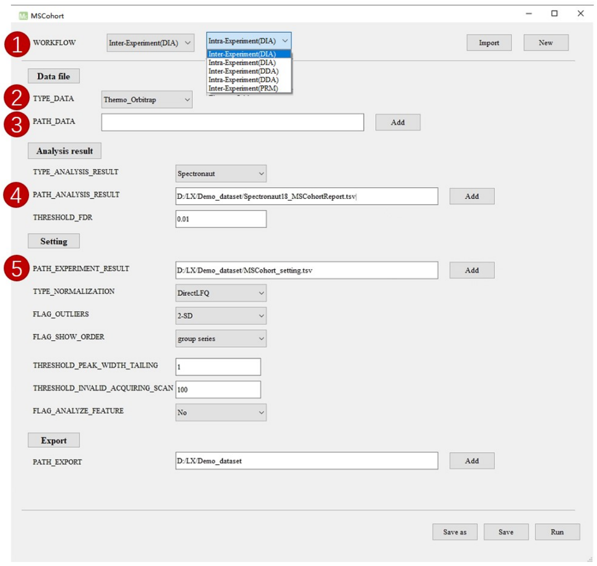

① Set **WORKFLOW** as **Inter-experiment(DIA)**;

② Select **TYPE_DATA** according to the data type ;

③ The **PATH_DATA** could be empty. MSCohort support the inter-experimental analysis mainly based on Spectronaut result, eliminating the need to submit raw files, which will obtain the result in a relatively **short time**. It is recommended for large cohort analysis.

In addition, users could choose to add the raw data. MSCohort would provide comprehensive analysis reports not only for inter-experiment, but also for intra-experiment analysis. This may **take a long time**.

④ Click Add to select the Spectronaut customized report for MSCohort into the **PATH_ANALYSIS_RESULT**;

⑤ Click Add to select the **MSCohort_setting.tsv** into the **PATH_EXPERIMENT_RESULT**;

MSCohort implements automated calculation of metrics for contaminants, retention time (RT) relative deviation, as well as the variations of identification/quantification results (the number of identifications, intensity distribution, Pearson correlation, etc.) between experiments. MSCohort reports corresponding scores to each of the inter-experiment metrics, and provides a heatmap overview, which yields an assessment of the quality at a glance and facilitates pinpointing the low-quality experiments.

MSCohort Inter-experiment Analysis Results

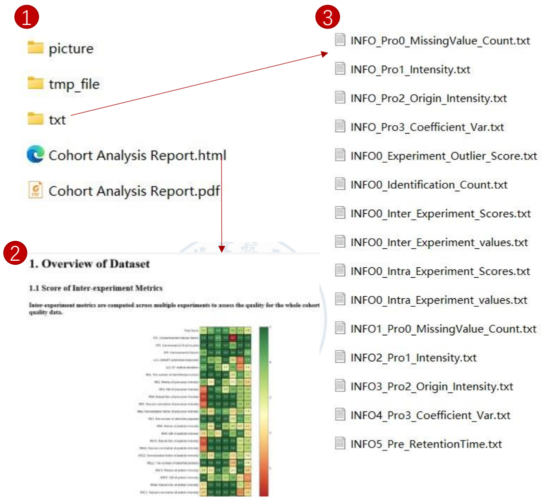

① The MSCohort results ;

② Double clicking **Cohort Analysis Report.html**, the report will be showed in the browser.

③ Double clicking **txt** folder, the outputs are also exported to simple tab -delimited text files.

23

Supplementary Note 4: Optimization of LC-MS Method for Establishment of SOP for Urinary Proteomics

For establishing a SOP, a well-performed LC-MS method is a prerequisite, to achieve high reproducibility, high throughput, and high sensitivity.

The LC-MS based proteomics workflow is complex and includes multiple procedures: sample preparation, liquid chromatography (LC) separation, and mass spectrometry (MS) acquisition. Many of the parameters and variations in these procedures are not independent but interact, making it challenging to establish an SOP with high sensitivity, high throughput, and high reproducibility. Some researchers consider LC-MS workflow as a "black box". The evaluation and optimization are performed by comparing the number of identified peptides and proteins of different LC-MS parameter combinations, which is time-consuming and laborious. For MS users, especially those without years of expertise, it is difficult to determine whether the experiment is successful in the number of identified peptides and proteins, whether an LC-MS parameter combination is applicable, and whether the LC-MS workflow is fully optimized according to specific biological and clinical samples.

MSCohort helps us optimize experimental parameters quickly and establish SOP by extracting comprehensive and systematic quality control (QC) metrics, establishing the relationship between QC metrics and identification results. Through a DIA scoring formula and comprehensive metrics analysis reports, it provides direct explanations for the underlying causes of data results. Users can determine appropriate parameter conditions through a few experiments and figure out whether additional experiments are needed based on MSCohort's reports, facilitating rapid optimization of experimental parameters and the establishment of an SOP.

Below, we present the process of optimizing urine proteomic acquisition methods based on the MSCohort quality control system, covering sample preparation, chromatographic separation, and mass spectrometry parameters (see Supplementary Methods for detailed parameters).

S.Note 4.1 Determining the Appropriate Sample Loading Amount

The sample loading amount is a critical variate of LC−MS workflow. A low loading amount leads to low peptide intensities and low identification rate, while an extremely high loading amount is impractical and even harmful to the instruments. Hence, choosing the optimal amount to achieve a balance between sample loading and identification depth is of great significance[22]. Under the guidance of the MSCohort QC system, we compared the results of different sample loading amounts (0.25, 0.5, 1, 2, 4, 8 μg) and determined the appropriate sample loading amount. As shown in Figure S.Note 4.1 a,b, we observed that the number of identified precursors, peptides and protein groups gradually increased from 0.25 μg to 2 μg, with the increase beginning to reach saturation above 2 μg.

The metrics reported in MSCohort and the DIA scoring formula can be used to illustrate the performance of the samples with different loading amounts. We choose the 0.5 μg data and 2 μg data for specific analysis. Figure S.Note 4.1 c,d showed that the acquired MS2 scans in 0.5 μg is closed to that in 2 μg data, while the identified MS2 scans and identified precursors are significantly increased in 2 μg data, which is also reflected in the increase of the identification rate of the MS2 scans (M19. MS2 identification rate) and the utilization rate of the MS2 scans (W5. Identified precursor/identified scan rate) in 2 μg data. Other metrics reported by MSCohort can be used to illustrate how the sample loading amount affects the identification rate and utilization rate of the MS2 scans. With the increase in sample loading from 0.25 μg to 2 μg, both peak intensity and peak counts of MS1 and MS2 gradually increased. Then, the identification rate of MS2 scans

increased from 0.61 to 0.71, and the spectra complexity of MS2 scans (W3. Redundant identified precursors/Identified scan rate) increased from 3.15 to 4.6. This means that each MS2 spectrum could identify more precursor ions, leading to an increase in the utilization rate of the MS2 scans from 0.82 to 1.23. At the same time, the acquired MS2 scans, the identification rate and utilization rate of the MS2 scans, and the spectra complexity of MS2 scans nearly do not increase above 2 μg (Figure S.Note 4.1 e). The above results indicate that a sample loading of 2 μg has achieved the optimal results under the current conditions.

In practice, the appropriate sample loading amount is not constant in different experiments. Users can explore the appropriate loading amount by performing a series of MS analyses and comparing the numbers of identified proteins, but there are some problems in this method such as long period, high cost, lack of validation, and so forth. The DIA scoring formula and the metrics extracted in MSCohort illustrate the relationship between loading amount and identification results. Users can determine the appropriate loading amount by performing a few experiments and figure out whether more experiments are necessary from the report of MSCohort.

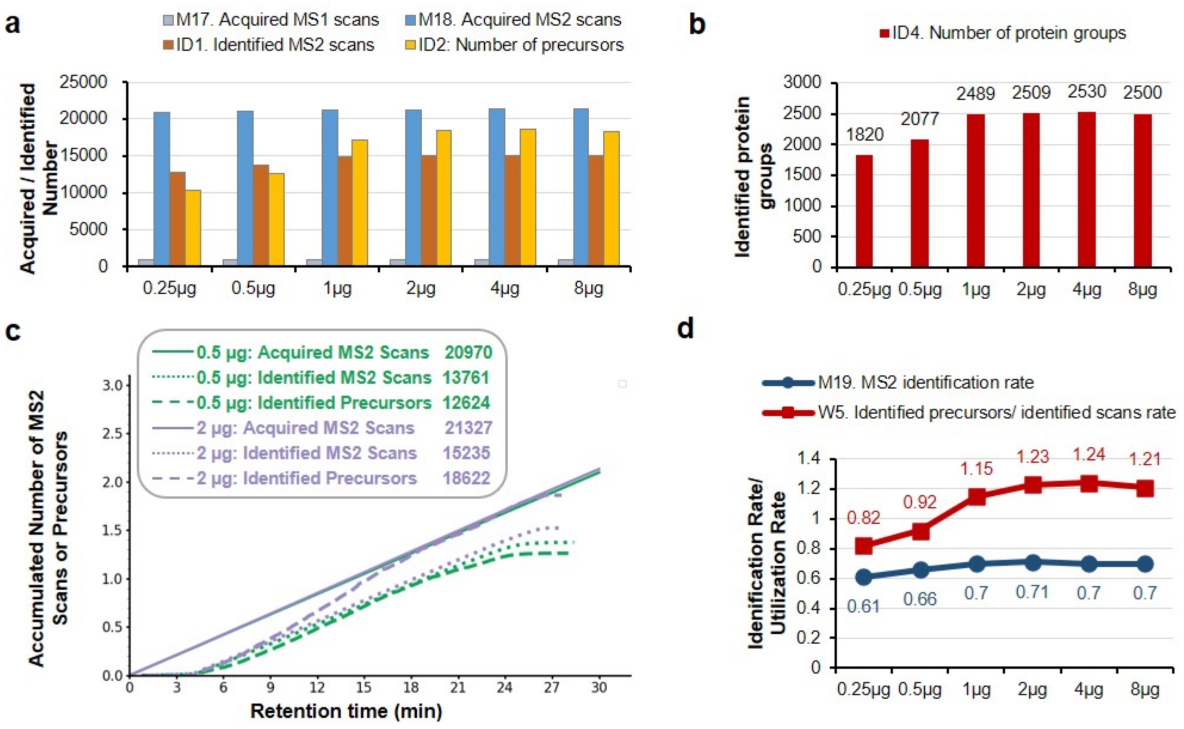

e

Sample Loading Amount	0.25 μg	0.5 μg	1 μg	2 μg	4 μg	8 μg
S3. Median total ion current (TIC)	8.53E+11	1.47E+12	3.21E+12	6.16E+12	9.20E+12	1.73E+13
M6. Median peaks intensity of MS1	3.92E+08	6.61E+08	1.49E+09	3.20E+09	4.81E+09	9.78E+09
M7. Median peak counts of MS1	1591.5	1724.5	1908	1994	2063.5	2033
M8. Median peak counts of MS2	433	517	605	669	755	771
M9. Median peaks intensity of MS2	8.87E+06	1.55E+07	3.22E+07	6.02E+07	9.05E+07	1.68E+08
M18. Acquired MS2 scans	20880	20970	21174	21263	21337	21439
M19. MS2 identification rate	0.61	0.66	0.7	0.71	0.7	0.7
W3. Redundant identified precursors/ Identified scan rate	3.15	3.55	4.33	4.6	4.67	4.61
W4. Redundant identified precursors/ Identified precursors Rate	3.86	3.87	3.76	3.75	3.77	3.79
W5. Identified precursors/ identified scans rate	0.82	0.92	1.15	1.23	1.24	1.21
ID1. Identified MS2 scans	12721	13761	14904	15235	15010	15037
ID2: Number of precursors	10409	12624	17158	18622	18577	18252
ID3: Number of peptides	8322	9970	13469	14205	14133	13775
ID4. Number of protein groups	1820	2077	2489	2509	2530	2500

Figure S.Note 4.1 The instrument performance under different sample loading amounts. **a** The number of acquired MS2 scans, identified MS2 scans, identified precursors, and identified peptides under different sample loading amounts. **b** The number of identified protein groups under different sample loading amounts. **c** Graph showing the accumulated number of MS2 scans or precursors of 0.5 and 2 μg over 30 min gradient time. **d** The identification rate (M19. MS2 identification rate) and utilization rate (W5. Identified precursor/identified scan rate) of MS2 under different sample loading amounts. **e** The mainly differential metrics in the MSCohort results.

S.Note 4.2 Improving the Appropriate Chromatographic Condition

Chromatographic separation is a key step in LC-MS based proteomics. Especially for large-scale urinary proteomics research, good separation efficiency, high throughput and reproducibility are necessary. Monolithic columns are becoming more and more popular among analytical scientists due to their high throughput. The porous structure and permeable nature of the monolithic column allows for rapid separation of complex mixtures, offering good separation efficiency and high peak capacity, while maintaining low back pressure to achieve efficiency and retention time stability in the separation process[23, 24, 25]. We optimized the chromatographic condition using a 50 cm × 50 μm monolithic silica capillary column.

Under the guidance of the MSCohort QC system, we determined the appropriate chromatographic flow rate by analyzing the performance under different chromatographic flow rates (300, 500 nL/min). As shown in Figure S.Note 4.2, we observed that the dead time decreased from 4.3 min to 2.45 min, with the flow rate increased from 300 to 500 nL/min. The reduction of chromatographic dead time significantly increases the effective acquiring time, thus improving the identification rate of the MS2 scans (M19. MS2 identification rate). At the same time, the metrics reported in MSCohort showed that there is no significant difference between 300nL/min and 500 nL/min on the number of identification precursors. Because the sensitivity is higher at the lower flow rate, so the spectra complexity of MS2 scans (W3. Redundant identified precursors/Identified scan rate) and the utilization rate of the MS2 scans (W5. Identified precursor/identified scan rate) is higher in 300 nL/min condition. And the identification rate of the MS2 scans is higher in 500 nL/min condition. In addition, we also collected 3 technical repetitions for quantitative repeatability analysis, and the results showed that 500 nL/min condition could achieve better quantitative precision than 300 nL/min condition. We also conducted continuous tests on 7 days with 500 nL/min flow rate, and the results showed that the chromatographic system achieved good retention time stability for 7 consecutive days (Figure S.Note 4.2 e), and the average retention time deviation was less than 0.25 min. Therefore, considering the qualitative and quantitative performance, the flow rate of 500 nL/min was selected.

MSCohort illustrates the relationship between the identification rate and utilization rate of the MS2 scans and identification results, as well as reports comprehensive metrics, providing the basis and reference for selecting suitable chromatographic conditions quickly.

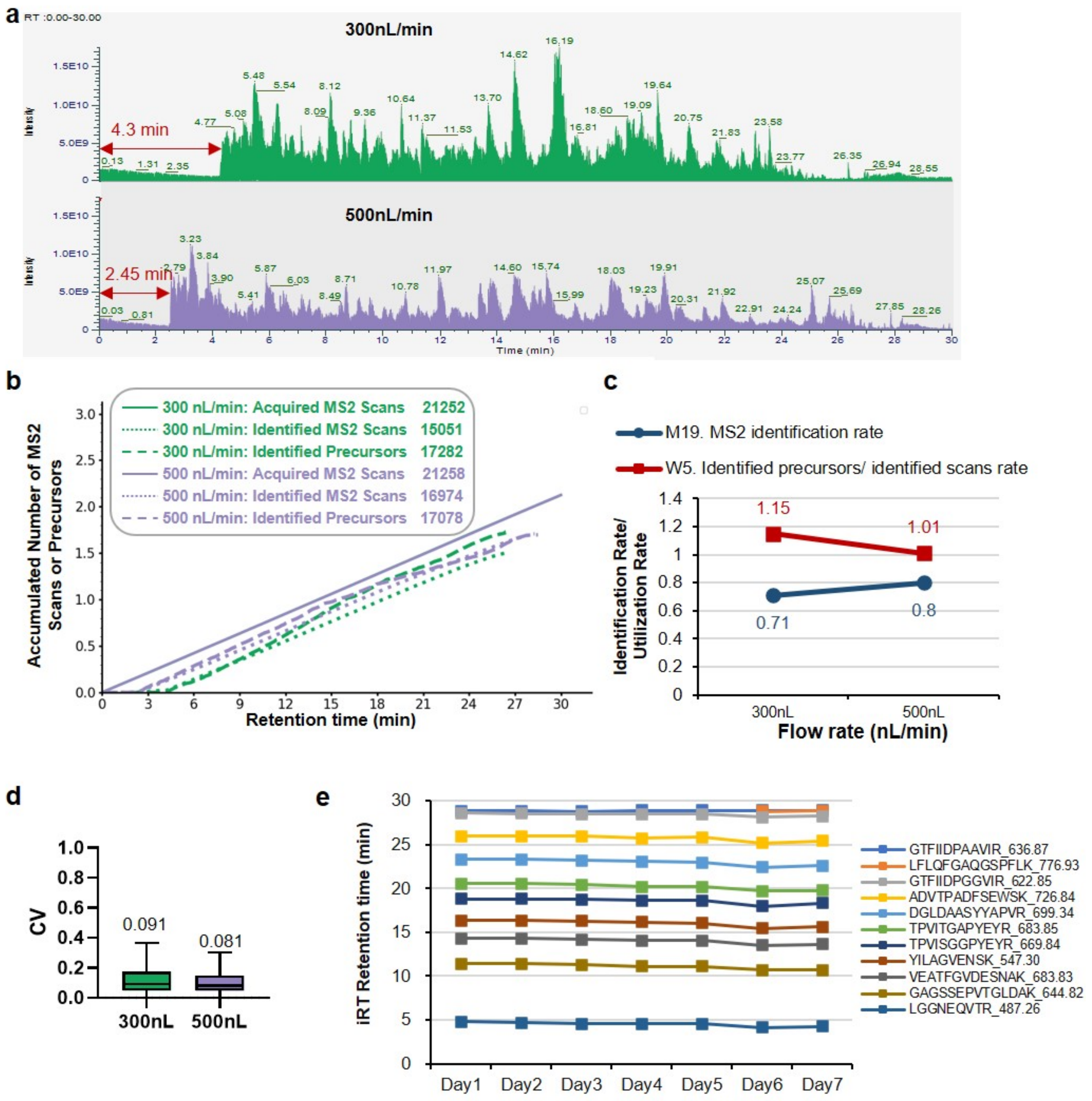

Figure S.Note 4.2 The instrument performance under different flow rates. **a** Initial time for peptides to elute out of the analytical column (dead time) under different flow rates. **b** Graph showing the accumulated number of MS2 scans or precursors for 300 nL/min and 500 nL/min over 30 min gradient time. **c** The identification rate (M19. MS2 identification rate) and utilization rate (W5. Identified precursor/identified scan rate) of MS2 under different flow rates. **d** The distribution of the coefficients of variation (CV) obtained on the protein intensity across the three technical replicates under different flow rates. The median CV was shown at the top of the graph. **e** The retention time of 11 iRT peptides over 7 consecutive days, the average retention time deviation was less than 0.25 min.

S.Note 4.3 Optimizing the Appropriate DIA windows

Previous studies have confirmed that the DIA method showed improved proteome coverage, reproducibility, and quantitative precision when compared with the label-free DDA method[26, 27, 28, 29]. Consequently, DIA is increasingly used for large-scale quantitative proteomics. Here, we optimized the DIA method for urinary proteomics based on MSCohort.

The setting of DIA windows is a key parameter that affects the identification and quantitative results of DIA. In DIA experiments, fragmentation spectra are highly complex due to the parallel fragmentation of multiple precursors. To reduce complexity, the range of precursor masses is distributed across multiple MS2 windows, which need to be designed by the experimenter. While increasing the number of MS2 windows results in less spectra complexity and higher proteome coverage, it comes at the expense of an increased cycle time, leading to fewer data points collected across the elution peak, impeding precise quantification. Achieving this balance poses a formidable challenge, necessitating context-specific optimization strategies tailored to factors such as sample complexity, abundance, and chromatography[6, 30].

MSCohort helps optimize this balance by extracting and reporting comprehensive metrics, as well as providing the relationship between the metrics and identification results based on the DIA scoring formula. Here, we utilize the method that involves multiple MS1 scans per duty cycle to strike a balance between reducing spectra complexity and increasing the number of data points per peak[6, 31, 32]. This decoupled scan event strategy ensured that a sufficient number of MS1 scan events could be acquired over the peptide chromatographic elution time to enable their precise quantification. At the same time, more MS2 windows were set in a way to improve peptide detection efficiency and proteome coverage. As shown in Figure S.Note 4.3, we observed a significant increase in the number of identified precursors, from 17078 to 21758 (a 27% increase), and in the number of identified protein groups, from 2403 to 3036 (a 26% increase), with the MS2 windows setting from 22 to 80. By comparing the results of different MS2 windows setting (40, 60, 80,100), 80 MS2 windows showed the highest identification results.

The metrics reported in MSCohort and the DIA scoring formula can be used to illustrate the performance of different MS2 windows settings. With the increase of DIA windows number, the cycle time increased and the precursors duplicate identification rate (W4. Redundant identified precursors/ Identified precursors rate) decreased, so the utilization rate of the MS2 scans (W5. Identified precursors/ identified scan rate) increased from 1.01 to 1.44, thus obtaining a higher identification depth. At the same time, with the increase of DIA windows number, the spectra complexity (W3. Redundant identified precursors/ Identified scan rate) decreased, and the identification rate of MS2 scans (M19. MS2 identification rate) decreased. Thus, the number of identified MS2 scans and identified precursors in 100 windows setting was decreased than that in 80 windows.

The identification rate of the MS2 scans, spectral complexity of MS2 scans, precursors duplicate identification rate, and the utilization rate of the MS2 scans in the DIA scoring formula visually explained the relationship between DIA windows and identification results, and helped users determine the appropriate DIA method. In addition, we also collected 3 technical repetitions for quantitative repeatability analysis, and the results showed that the coefficients of variation (CV) of 80 windows were comparable to 22 windows (Figure S.Note 4.3 f).

The above experimental optimization is the process of optimizing the urinary proteomics method and establishing SOP on Orbitrap Exploris 480 based on the MSCohort QC system. While, the timsTOF implements a novel diaPASEF (parallel accumulation–serial fragmentation combined with data-independent acquisition) scan mode[33], and we also optimized DIA method for timsTOF

data collection based on MSCohort. The sample loading amount was 500 ng, the chromatographic conditions were consistent with Orbitrap Exploris 480, and the number of DIA windows was 50 (Supplementary Data 2, Supplementary Note 3).

Taken together, we systematically optimized the LC-MS method for urinary proteomics based on MSCohort, and developed a high sensitivity, high throughput, and high reproducibility DIA-based SOP for urinary proteomics analysis. More than 3000 protein groups could be identified in a single run of 30 minutes, representing the most comprehensive coverage of the urinary proteome achieved in a short gradient (less than 1 hour) to date.

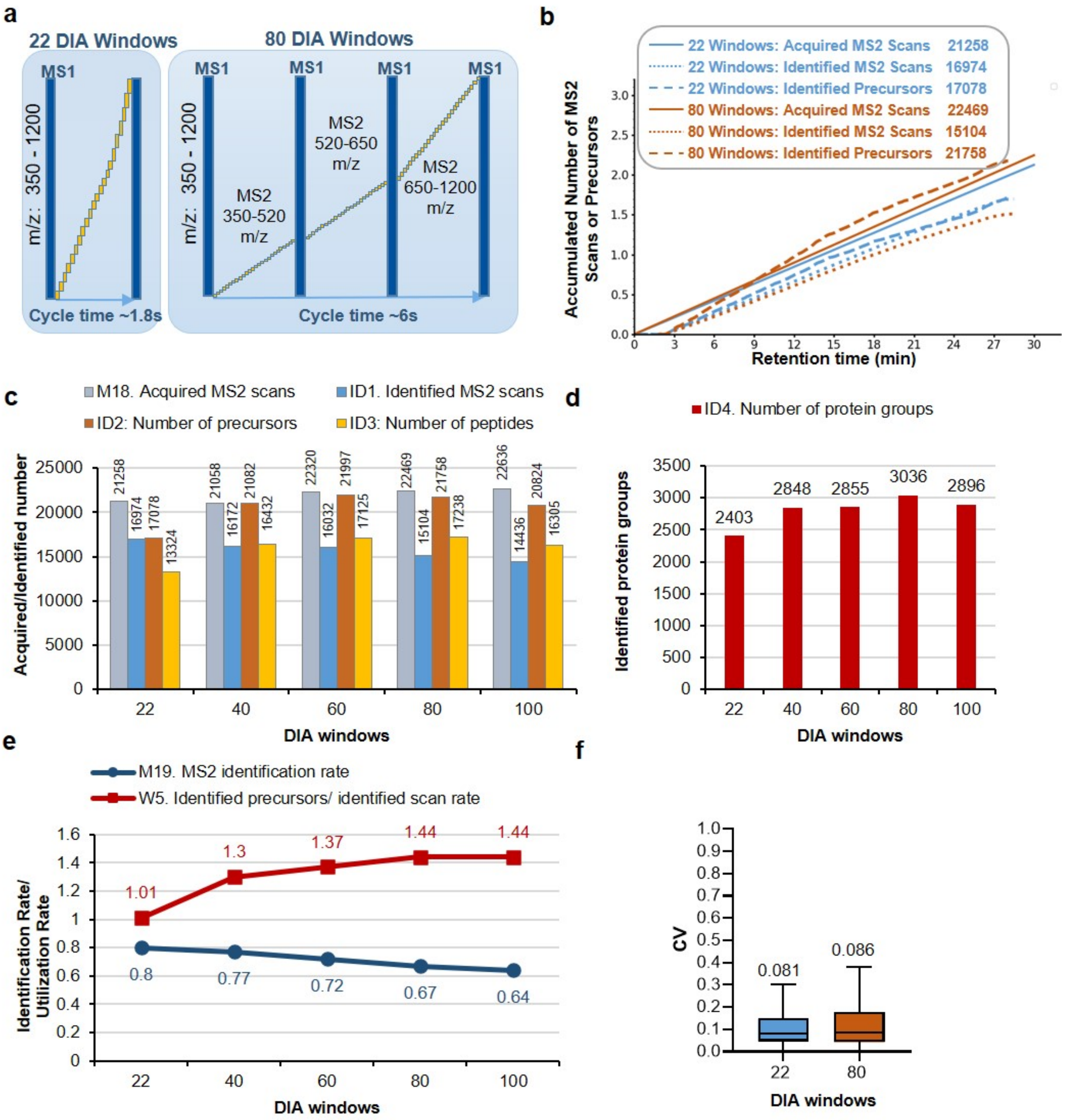

g

DIA windows number	22	40	60	80	100
W6. MS1 data point per peak	4	6	4	6	6
W7. MS2 data point per peak	4	3	2	2	1
M4. MS1 cycle time(s)	1.85	1.7	2.43	2.09	2.63
M5. MS2 cycle time(s)	1.85	3.43	4.85	6.4	7.96
M18. Acquired MS2 scans	21258	21058	22320	22469	22636
M19. MS2 identification rate	0.8	0.77	0.72	0.67	0.64
W3. Redundant identified precursors/ Identified scan rate	4.1	3.21	2.6	2.27	2.06
W4. Redundant identified precursors/ Identified precursors rate	4.07	2.47	1.89	1.58	1.43
W5. Identified precursors/ identified scans rate	1.01	1.3	1.37	1.44	1.44
ID1. Identified MS2 scans	16974	16172	16032	15104	14436
ID2: Number of precursors	17078	21082	21997	21758	20824
ID3: Number of peptides	13324	16432	17125	17238	16305
ID4. Number of protein groups	2403	2848	2855	3036	2896

Figure S.Note 4.3 The instrument performance under different DIA windows setting. **a** Diagrams

of a duty cycle with a single MS1 survey scan (22 DIA Windows) and a duty cycle with three MS1 survey scans (80 DIA Windows). **b** Graph showing the accumulated number of MS2 scans or precursors for 22 DIA Windows and 80 DIA Windows over 30 min gradient time. **c** The number of acquired MS2 scans, identified MS2 scans, identified precursors, and identified peptides under different DIA windows setting. **d** The number of identified protein groups in different DIA windows setting. **e** The identification rate (M19. MS2 identification rate) and utilization rate (W5. Identified precursor/identified scan rate) of MS2 under different sample loading amounts. **f** The distribution of the coefficients of variation (CV) obtained on the protein intensity across the three technical replicates under different DIA settings. The median CV was shown at the top of the graph. **g** The mainly differential metrics in the MSCohort results.

Supplementary Note 5: The Novelty of Our Developed Standard Operating Procedure (SOP) for Urinary Proteomics

For large-scale urine proteomics studies, there is a pressing need for robust, high-throughput, and highly reproducible SOP. The sample preparation procedure requires an efficient, high-throughput, and high reproducible protocol to guarantee the comparability in sample quality. The LC system should exhibit good separation efficiency to facilitate the effective separation of complex protein mixtures in urine, along with robust retention time stability and reproducibility to ensure reproducible separation outcomes across different samples. The mass spectrometry system requires high throughput and high sensitivity to achieve comprehensive identification and accurate quantification of extensive urinary proteomes. Currently, there is a lack of SOP for urinary proteomics. Each LC-MS platform applied its own experiment and data acquisition procedures, which resulted in a large variation across instruments, platforms, and laboratories[15, 34, 35, 36]. Therefore, it is necessary to establish a unified, high-throughput, high-reproducible SOP.

To meet the above requirements, we developed an SOP for urinary proteomics that combines the following advantages:

(1) In the sample preparation procedure, we have developed a high-throughput urine sample preparation method, called 96DRA-Urine (Direct reduction/alkylation in urine)[20], based on PVDF membrane, which possesses the following characteristics: First, proteins in urine are directly subjected to reduction and alkylation before acetone precipitation. Post-reduction and alkylation, protein disulfide bonds are cleaved, leading to improved precipitation efficiency and enhanced protein identification depth. Additionally, the precipitated proteins dissolve more readily in Tris. This method eliminates the need for denaturants or detergents such as urea and SDS, reducing the proportion of unexpectedly modified peptides and contamination in mass spectrometry. Second, using a 96-well PVDF plate has reduced buffer exchange time, thereby increasing sample preparation throughput. Within 6 hours, rapid preprocessing of 96 samples is achievable, with actual manual handling time less than 3 hours. One person can complete the preparation of at least two 96-well plates in a day, equating to processing approximately 200 samples per day. Third, our previous experiments demonstrate that compared to the in-solution, FASP, and MStern methods, this approach achieves higher identification depth while maintaining superior experimental reproducibility[20]. Fourth, we optimized the sample loading amount to achieve high proteome coverage. Altogether, our 96DRA-Urine method enables efficient, high-throughput, sensitive, and reproducible urine sample preparation.

(2) In the liquid chromatography (LC) separation procedure, we employ a 50 cm × 50 μm monolithic silica capillary column and optimized the chromatographic condition (see Supplementary Note 4 for details), which possesses the following characteristics: First, the porosity and permeability of the monolithic column enable rapid separation of complex mixtures, exhibiting excellent separation efficiency and high peak capacity. Under the guidance of the MSCohort QC system, we have optimized the chromatographic gradient settings to reduce dead time, achieving extended effective separation gradients and enhanced quantitative reproducibility[23, 24, 25]. Second, another advantage of the monolithic column is its low back pressure (at a flow rate of 500 nL/min, the pressure is only 220 bar for a 50 cm × 50 μm analytical column), which facilitates efficient separation and retention time stability. This character is particularly advantageous during long-term LC-MS data acquisition as it ensures comparability of retention times between different samples and stable ESI spraying over prolonged periods, making it especially suitable for high-throughput

large-scale proteomic analyses[37, 38].

(3) In the mass spectrometry procedure, we optimized the data-independent acquisition (DIA) method for urinary proteomics that achieves high throughput and deep identification based on the MSCohort QC system and DIA scoring formula. As described in Supplementary Note 4, the setting of DIA windows number is a crucial step that influences DIA results. Striking a balance between achieving identification depth and quantitative accuracy through the setting of DIA windows number is a complex issue. Under the guidance of the MSCohort QC system and DIA scoring formula, after two rounds of optimization, the results demonstrate that the optimized DIA acquisition method with 80 DIA windows increased the number of identified precursors by 27% and identified proteins by 26%, leading to enhanced MS2 identification depth and MS1 quantitative reproducibility. Our results showed that within 30 minutes, over 3000 urine protein groups were identified in a single sample, representing the highest single-run urine proteome identification depth under short gradient (30 min) conditions to date.

(4) In the quality evaluation procedure, we developed the comprehensive MSCohort QC system. MSCohort consists of two modules: Intra-experiment analysis and inter-experiment analysis. It can apply not only to process evaluation and optimization of individual experiments, but also to process quantitative assessment of system performance across multiple experiments. In practice, after each sample was collected, the quality of individual experiment data could be assessed using MSCohort intra-experiment analysis, assisting users in monitoring the whole LC-MS workflow performance, promptly identifying data quality problems, and guiding to trigger troubleshooting when necessary. After all data collection is completed, inter-experiment analysis using MSCohort could be performed to comprehensively evaluate the stability and reproducibility of data acquisition across multiple experiments, including retention time stability, Pearson correlation, quantitative precision, etc. MSCohort also incorporates unsupervised machine learning algorithm (isolation forest) to detect potential outlier experiments. Altogether, MSCohort is a comprehensive quality control system (tool) to ensure reproducibility and robustness during data generation and maximize the accessibility of downstream data.

Taken together, in this study, we developed a SOP for urinary proteomics (Supplementary Note 3). We think that this SOP is currently worth learning for various LC-MS platforms as it integrates the optimal strategies at each step, including the 96DRA-Urine high-throughput sample processing method, stable and efficient chromatography system, high sensitive and high-throughput DIA-based MS method, and comprehensive MSCohort QC system performed systematic quality evaluation and monitor during the acquisition process. We applied this SOP to urinary proteomics studies across multiple LC-MS platforms, and the results demonstrate that with the combination of comprehensive QC system and unified SOP, multiple LC-MS platforms achieved robust, highly sensitive, and highly reproducible data generation (Fig. 4), facilitating the transition of urinary proteomics into clinical proteomic research practice.

Supplementary Note 6: Comparative analysis of urine proteome DDA data from multi-platform study

Additionally, we also conducted DDA experiments on 20 LC-MS platforms to assess the data reproducibility under conditions with SOP and without SOP.

We analyzed DDA data for 20 LC-MS platforms. Within the collection time of 30 minutes, the qualitative number and consistency of urine DDA data were significantly lower than that of DIA data under the same condition. The range of identified proteins varied from 1020 to 2186 among the 10 platforms without LC-SOP and 1348 to 2402 among the 10 platforms with LC-SOP (Supplementary Figure 15). Although the consistency of identification results have been improved after employing SOP, the consistency of DDA is still lower than that of DIA due to stochastic MS2 sampling[39]. Generally, DDA data results indicate that timsTOF data showed better performance in the depth of protein identification, due to the enhanced sensitivity of the instrument implementing a novel PASEF scan mode[40, 41, 42]. While for DIA data, there is relatively little difference between Orbitrap and timsTOF instruments under the same LC condition.

We also analyze the precision and reproducibility of quantitative results for DDA data. The median CV for each LC-MS platform was below 20% and the Pearson correlation coefficients were greater than 0.93, demonstrating the high quantitative precision and reproducibility of DDA data at the intra-instrument level. In addition, the Pearson correlation among different platforms was comparable to DIA data. Furthermore, low levels of carry-over were observed for 20 LC-MS platforms (<1.5%), indicating the good performance of current LC systems (Supplementary Figure 16).

Taken together, the above results demonstrate that the consistency of identification results of DDA data across multi-platform is lower than that of DIA data in the same condition.

Supplementary Methods

1. Acquisition and processing of the optimization dataset

The urine quality control peptide samples were prepared by the 96 DRA-Urine method as Methods described. LC-MS analysis of the optimization dataset was collected by Orbitrap Exploris 480 mass spectrometer coupled with Vanquish Neo UHPLC system (Thermo Fisher Scientific). All data acquisition was operated in DIA mode over a 30-minute total gradient.

For optimization of the sample loading amount dataset, 0.25, 0.5, 1, 2, 4, and 8 μg peptides were loaded, respectively, and separated at a constant flow rate of 300 nL/min by analytical column (50cm × 50μm monolithic silica capillary column (Beijing Uritech Biotech)). LC mobile phases A and B were 100% H_2O with 0.1% FA (v/v) and 80% ACN / 20% H_2O with 0.1% FA (v/v), respectively. In 30 min experiments, the gradient of mobile phase B increased from 5% to 20% over 22 min and then increased to 30% over 3 min, a further 1 min plateau phase at 90% B, and a 4 min wash phase of 1% B. Data was acquired in DIA mode using 22 variable windows covering a mass range of 350–1200 m/z. As a basis, we took the previously optimized DIA method as reference[30]. The resolution was set to 120,000 for MS1 and 30,000 for MS2. The Normalized AGC Target was 300% for MS1 and 200% for MS2, with a maximum injection time of 50 ms in MS1 and 50 ms in MS2. HCD Normalized Collision Energies was set to 30%.

For optimization of the chromatographic condition dataset, 2 μg peptide was loaded, respectively, and separated at a flow rates of 300, 500 nL/min by analytical column (50cm × 50μm monolithic silica capillary column (Beijing Uritech Biotech)). LC mobile phases A and B were 100% H_2O with 0.1% FA (v/v) and 80% ACN / 20% H_2O with 0.1% FA (v/v), respectively. In 30 min experiments, the gradient of mobile phase B increased from 5% to 20% over 22 min and then increased to 30% over 3 min, a further 1 min plateau phase at 90% B, and a 4 min wash phase of 1% B. Data was acquired in DIA mode using 22 variable windows covering a mass range of 350–1200 m/z. The resolution was set to 120,000 for MS1 and 30,000 for MS2. The Normalized AGC Target was 300% for MS1 and 200% for MS2, with a maximum injection time of 50 ms in MS1 and 50 ms in MS2. HCD Normalized Collision Energies was set to 30%.

For optimization of the DIA windows dataset, 2 μg peptide was loaded, respectively, and separated at a flow rate of 500 nL/min by analytical column (50cm × 50μm monolithic silica capillary column (Beijing Uritech Biotech)). LC mobile phases A and B were 100% H_2O with 0.1% FA (v/v) and 80% ACN / 20% H_2O with 0.1% FA (v/v), respectively. In 30 min experiments, the gradient of mobile phase B increased from 5% to 20% over 22 min and then increased to 30% over 3 min, a further 1 min plateau phase at 90% B, and a 4 min wash phase of 1% B. Data was acquired in DIA mode using 40, 60, 80, 100 variable windows covering a mass range of 350–1200 m/z. For the method with increased MS1 sampling, a second and third MS1 scan were inserted after one-third of the total MS2 scan. The resolution was set to 120,000 for MS1 and 30,000 for MS2. The Normalized AGC Target was 300% for MS1 and 200% for MS2, with a maximum injection time of 50 ms in MS1 and 50 ms in MS2. HCD Normalized Collision Energies was set to 30%.

The DIA data for all optimization datasets were analyzed separately with Spectronaut v.19.0 performing the directDIA analysis. Default settings were used unless otherwise noted. Cross-run normalization was turned off. Carbamidomethyl on C was set as fixed modification, and Oxidation on M as variable modification. All searches were performed against the human SwissProt database (Homo sapiens, 20,386 reviewed entries, 2022_06 version), concatenated with iRT peptide.fasta file. The data processing results were exported using customized reports for further data analysis using MSCohort.

2. Acquisition of DDA data from 20 LC-MS platforms

The 20 participant platforms used 3 different types of mass spectrometers, including Orbitrap (ThermoFisher Scientific), timsTOF (Bruker Daltonik), and ZenoTOF (SCIEX). To systematically analyze the variation among different LC-MS platforms and the main influencing factors, we divided 20 platforms into 2 groups. Ten of these platforms used the procedures and parameters they routinely used (number M01-M10), and the other 10 LC-MS platforms employed a unified LC condition (the same type of column and same gradient) and consistent MS parameters for the same type of instrument (number U01-U10).

DDA was performed on the same urine QC sample and using the same LC gradient as in the DIA runs. The detailed LC and MS acquisition parameters are provided in Supplementary Data 2, and the Standard Operating Procedure is provided in Supplementary Note 1. All participant LC-MS platforms collected 1 pure iRT (Biognosys) data, 3 DDA data, 3 DIA data, and 1 blank to assess carry-over. The acquisition time was 30 min. The performance of DDA data acquired across multi-platform was investigated and provided in Supplementary Figure 5.

3. Processing of DDA data from 20 LC-MS platforms

Data-dependent acquisition data from the 20 participant platforms were processed using Proteome Discoverer (version 2.4, Thermo Fisher Scientific) software and searched against the human SwissProt database (Homo sapiens, 20386 reviewed entries, 2022_06 version). The .d and .wiff format files were converted into .mgf format using the MSconvert tool from the ProteoWizard (version 3.0.22317). The following Sequest HT search parameters were used: parent ion mass tolerance of 10 ppm, fragment ion mass tolerance of 0.02 Da; carbamidomethyl of cysteine was set as a fixed modification and oxidation (M) as variable modification. Proteins were positively identified if they had an FDR of less than 1.0% and at least two unique peptides.

The quantitative analysis was processed using MaxQuant (version 2.2.0.0). All searches were performed against the human SwissProt database (Homo sapiens, 20386 reviewed entries, 2022_06 version). MaxQuant parameters were set as follows: search type, Standard for Orbitrap and ZenoTOF DDA data, TIMS-DDA for timsTOF ddaPASEF data; Carbamidomethyl on C as fixed modification; Oxidation on M and Acetyl at protein N-terminus as variable modifications; the specific enzyme used Trypsin/P and the number of maximum missed cleavages was set to 2; peptide mass tolerance in first search, 20 ppm; peptide mass tolerance in the main search, 4.5 ppm for Orbitrap data and 10 ppm for timsTOF data and ZenoTOF data; PSM and protein FDR were both set to 0.01. Label-free quantification was performed in MaxQuant for protein quantitation. The data processing results were exported and further processed quality control analysis using MSCohort.

References

1. Rudnick, P.A., et al. Performance metrics for liquid chromatography-tandem mass spectrometry systems in proteomics analyses. *Mol. Cell. Proteomics* **9**, 225-241 (2010).
2. Ma Z., et al. QuaMeter: multivendor performance metrics for LC-MS/MS proteomics instrumentation. *Anal. Chem.* **84**, 5845-5850 (2012).
3. Wang X., Chambers M.C., Vega-Montoto L.J., Bunk D.M., Stein S.E., Tabb D.L. QC metrics from CPTAC raw LC-MS/MS data interpreted through multivariate statistics. *Anal. Chem.* **86**, 2497-2509 (2014).
4. Morgenstern D., Barzilay R., Levin Y. RawBeans: A Simple, Vendor-Independent, Raw-Data Quality-Control Tool. *J. Proteome Res.* **20**, 2098-2104 (2021).
5. Huffman R.G., Chen A., Specht H., Slavov N. DO-MS: Data-Driven Optimization of Mass Spectrometry Methods. *J. Proteome Res.* **18**, 2493-2500 (2019).
6. Wallmann G., Leduc A., Slavov N. Data-Driven Optimization of DIA Mass Spectrometry by DO-MS. *J. Proteome Res.* **22**, 3149-3158 (2023).
7. Bielow C., Mastrobuoni G., Kempa S. Proteomics Quality Control: Quality Control Software for MaxQuant Results. *J. Proteome Res.* **15**, 777-787 (2016).
8. Chiva C., et al. QCloud: A cloud-based quality control system for mass spectrometry-based proteomics laboratories. *PLoS One* **13**, e0189209 (2018).
9. Olivella R., et al. QCloud2: An Improved Cloud-based Quality-Control System for Mass-Spectrometry-based Proteomics Laboratories. *J. Proteome Res.* **20**, 2010-2013 (2021).
10. Stanfill B.A., et al. Quality Control Analysis in Real-time (QC-ART): A Tool for Real-time Quality Control Assessment of Mass Spectrometry-based Proteomics Data. *Mol. Cell. Proteomics* **17**, 1824-1836 (2018).
11. Dogu E., et al. MSstatsQC 2.0: R/Bioconductor Package for Statistical Quality Control of Mass Spectrometry-Based Proteomics Experiments. *J. Proteome Res.* **18**, 678-686 (2019).
12. QuiC 5: Quality Control Monitoring in the Blink of an Eye. https://biognosys.com/content/uploads/2023/10/UserManual__QuiC5.pdf. (2023).
13. Tang M., et al. Comprehensive Evaluation and Optimization of the Data-Dependent LC-MS/MS Workflow for Deep Proteome Profiling. *Anal. Chem.* **95**, 7897-7905 (2023).
14. Geyer, P.E., et al. Plasma Proteome Profiling to detect and avoid sample-related biases in biomarker studies. *EMBO Mol. Med.* **11**, e10427 (2019).
15. Virreira Winter, S., et al. Urinary proteome profiling for stratifying patients with familial Parkinson’s disease. *EMBO Mol. Med.* **13**, e13257 (2021).
16. Guo, Z., et al. Analysis of the differential urinary protein profile in IgA nephropathy patients of Uygur ethnicity. *BMC Nephrol.* **19**, 358 (2018).
17. Cox, J. & Mann, M. MaxQuant enables high peptide identification rates, individualized p.p.b.-range mass accuracies and proteome-wide protein quantification. *Nat. Biotechnol.* **26**, 1367-1372 (2008).
18. Bittremieux W., Valkenborg D., Martens L., Laukens K. Computational quality control tools for mass spectrometry proteomics. *Proteomics* **17**, 1600159 (2017).
19. Jiang, Y., et al. Comprehensive Overview of Bottom-Up Proteomics using Mass Spectrometry. *ArXiv* (2023).
20. Tang, X., et al. 96DRA-Urine: A high throughput sample preparation method for urinary proteome analysis. *J. Proteomics* **257**, 104529 (2022).
21. Sun W., et al. Microwave-assisted protein preparation and enzymatic digestion in proteomics.

Mol. Cell. Proteomics **5**, 769-776 (2006).
22. Huang P., Liu C., Gao W., Chu B., Cai Z., Tian R. Synergistic optimization of Liquid Chromatography and Mass Spectrometry parameters on Orbitrap Tribrid mass spectrometer for high efficient data-dependent proteomics. *J. Mass Spectrom.* **56**, e4653 (2021).
23. Horie K., et al. Hydrophilic interaction chromatography using a meter-scale monolithic silica capillary column for proteomics LC-MS. *Anal. Chem.* **86**, 3817-3824 (2014).
24. Urisman A., et al. An Optimized Chromatographic Strategy for Multiplexing In Parallel Reaction Monitoring Mass Spectrometry: Insights from Quantitation of Activated Kinases. *Mol. Cell. Proteomics* **16**, 265-277 (2017).
25. Iwasaki M., Sugiyama N., Tanaka N., Ishihama Y. Human proteome analysis by using reversed phase monolithic silica capillary columns with enhanced sensitivity. *J. Chromatogr. A* **1228**, 292-297 (2012).
26. Muntel J., et al. Advancing Urinary Protein Biomarker Discovery by DataIndependent Acquisition on a Quadrupole-Orbitrap Mass Spectrometer. *J. Proteome Res.* **14**, 4752-4762 (2015).
27. Bruderer, R., et al. Extending the limits of quantitative proteome profiling with data-independent acquisition and application to acetaminophen-treated three-dimensional liver microtissues. *Mol. Cell. Proteomics* **14**, 1400-1410 (2015).
28. Selevsek N., et al. Reproducible and consistent quantification of the Saccharomyces cerevisiae proteome by SWATH-mass spectrometry. *Mol. Cell. Proteomics* **14**, 739-749 (2015).
29. Tüshaus J., et al. A region-resolved proteomic map of the human brain enabled by high-throughput proteomics. *EMBO J.* **42**, e114665 (2023).
30. Bruderer R., et al. Optimization of Experimental Parameters in Data-Independent Mass Spectrometry Significantly Increases Depth and Reproducibility of Results. *Mol. Cell. Proteomics* **16**, 2296-2309 (2017).
31. Xuan, Y., et al. Standardization and harmonization of distributed multi-center proteotype analysis supporting precision medicine studies. *Nat. Commu.* **11**, 5248 (2020).
32. Prakash, A., et al. Hybrid Data Acquisition and Processing Strategies with Increased Throughput and Selectivity. *J. Proteome Res.* **13**, 5415-5430 (2014).
33. Meier F., et al. diaPASEF: parallel accumulation–serial fragmentation combined with data-independent acquisition. *Nat. Methods* **17**, 1229-1236 (2020).
34. Sun, Y., et al. Noninvasive urinary protein signatures associated with colorectal cancer diagnosis and metastasis. *Nat. Commu.* **13**, 2757 (2022).
35. Tian, W., et al. Immune suppression in the early stage of COVID-19 disease. *Nat. Commu.* **11**, 5859 (2020).
36. Bi, X., et al. Proteomic and metabolomic profiling of urine uncovers immune responses in patients with COVID-19. *Cell Rep.* **38**, 110271 (2021).
37. Rigobello-Masini M., Penteado J.C., Masini J.C. Monolithic columns in plant proteomics and metabolomics. *Anal. Bioanal. Chem*. **405**, 2107-2122 (2013).
38. Hemdan A., Abdel-Aziz O. Application of a Fast Separation Method for Anti-diabetics in Pharmaceuticals Using Monolithic Column: Comparative Study With Silica Based C-18 Particle Packed Column. *J. Chromatogr. Sci.* **56**, 351-357 (2018).
39. Tabb D.L., et al. Repeatability and reproducibility in proteomic identifications by liquid chromatography-tandem mass spectrometry. *J. Proteome Res.* **9**, 761-776 (2010).
40. Meier, F. et al. Parallel Accumulation-Serial Fragmentation (PASEF): Multiplying Sequencing Speed and Sensitivity by Synchronized Scans in a Trapped Ion Mobility Device. *J. Proteome Res.* **14**, 5378-5387 (2015).

41. Meier, F. et al. Online Parallel Accumulation-Serial Fragmentation (PASEF) with a Novel Trapped Ion Mobility Mass Spectrometer. *Mol. Cell. Proteomics* **17**, 2534-2545 (2018).

42. Prianichnikov, N. et al. MaxQuant Software for Ion Mobility Enhanced Shotgun Proteomics. *Mol. Cell. Proteomics* **19**, 1058-1069 (2020).
