# Supplementary material for: Standard operating procedure combined with comprehensive quality control system for multiple LC-MS platforms urinary proteomics
Source: Nat Commun. 2025 Jan 26;16:1051. doi: 10.1038/s41467-025-56337-4 (PMC11770173; doi:10.1038/s41467-025-56337-4)
Supplement: Supplementary file 2 — Reporting Summary [file 41467_2025_56337_MOESM2_ESM.pdf]

nature portfolio

Corresponding author(s): Chao Liu, Wei Sun

Last updated by author(s): Dec 21, 2024

Reporting Summary

Nature Portfolio wishes to improve the reproducibility of the work that we publish. This form provides structure for consistency and transparency in reporting. For further information on Nature Portfolio policies, see our Editorial Policies and the Editorial Policy Checklist.

Statistics

For all statistical analyses, confirm that the following items are present in the figure legend, table legend, main text, or Methods section.

n/a	Confirmed	
☐	☒	The exact sample size (*n*) for each experimental group/condition, given as a discrete number and unit of measurement
☐	☒	A statement on whether measurements were taken from distinct samples or whether the same sample was measured repeatedly
☐	☒	The statistical test(s) used AND whether they are one- or two-sided<br>*Only common tests should be described solely by name; describe more complex techniques in the Methods section.*
☐	☒	A description of all covariates tested
☐	☒	A description of any assumptions or corrections, such as tests of normality and adjustment for multiple comparisons
☐	☒	A full description of the statistical parameters including central tendency (e.g. means) or other basic estimates (e.g. regression coefficient) AND variation (e.g. standard deviation) or associated estimates of uncertainty (e.g. confidence intervals)
☐	☒	For null hypothesis testing, the test statistic (e.g. *F*, *t*, *r*) with confidence intervals, effect sizes, degrees of freedom and *P* value noted<br>*Give P values as exact values whenever suitable.*
☒	☐	For Bayesian analysis, information on the choice of priors and Markov chain Monte Carlo settings
☒	☐	For hierarchical and complex designs, identification of the appropriate level for tests and full reporting of outcomes
☐	☒	Estimates of effect sizes (e.g. Cohen's *d*, Pearson's *r*), indicating how they were calculated

Our web collection on statistics for biologists contains articles on many of the points above.

Software and code

Policy information about availability of computer code

Data collection	All Orbitrap instrument data were collected by Xcalibur, which is commercial software by Thermo Fisher Scientific. All timsTOF instrument data were collected by timsControl 4.0, which is commercial software by Bruker. All ZenoTOF 7600 data were collected by SCIEX OS 3.0, which is commercial software by SCIEX.
Data analysis	The commercial software Spectronaut (version 18.0) was used to perform protein identification and quantification for DIA data. The commercial software Proteome Discoverer (version 2.4, Thermo Fisher Scientific) was used to perform protein identification for DDA data. MaxQuant (version 2.2.0.0) was used to perform protein quantification for DIA data. LIMMA package (version 3.58) in R (version 4.3) was used to perform differentially expressed proteins analysis. The commercial software Ingenuity Pathway Analysis (Qiagen) was used for pathway analysis. The correlation and t-SNE plots were performed using Corrplot (version 0.92) and Rtsne (version 0.16) packages in R (version 4.3). Enrich GO terms and KEGG pathways analysis were performed using clusterProfiler (version 4.8.3) in R (version 4.3). Pattern recognition analysis (OPLS-DA) was performed using SIMCA 14.0 (Umetrics, Sweden) software. Machine learning models (Logistic Regression, K-Nearest Neighbor, Gaussian Naïve Bayes, Support Vector Machines, Random Forest, Gradient Boosting Decision Tree) were performed using scikit-learn modules (version 0.23) in Python (version 3.7). Protein–protein interaction (PPI) plot was performed using STRING (version 11.0) and Cytoscape (version 3.7.2).

For manuscripts utilizing custom algorithms or software that are central to the research but not yet described in published literature, software must be made available to editors and reviewers. We strongly encourage code deposition in a community repository (e.g. GitHub). See the Nature Portfolio guidelines for submitting code & software for further information.

Data

Policy information about availability of data

All manuscripts must include a data availability statement. This statement should provide the following information, where applicable:

- Accession codes, unique identifiers, or web links for publicly available datasets
- A description of any restrictions on data availability
- For clinical datasets or third party data, please ensure that the statement adheres to our policy

The mass spectrometry proteomics data and search results have been deposited to the ProteomeXchange Consortium (https://proteomecentral.proteomexchange.org) via the iProX partner repository with the dataset identifier PXD050291 (in ProteomeXchange) and IPX0008194000 (in iProX).

Research involving human participants, their data, or biological material

Policy information about studies with human participants or human data. See also policy information about sex, gender (identity/presentation), and sexual orientation and race, ethnicity and racism.

Reporting on sex and gender	A total of 80 colorectal cancer (CRC) patients (48 males and 32 females; median age 57 years, min-max: 42–69 years) were recruited from the Cancer Hospital, Chinese Academy of Medical Sciences. All patients were pathologically diagnosed by two senior pathologists, and first-morning midstream urine samples were collected before surgical operations or chemotherapy/radiotherapy. In addition, 80 urine samples from healthy control (HC) (52 males and 28 females; median age 55 years, min-max: 40–68 years) were obtained from the Health Medical Center of the Cancer Hospital.
Reporting on race, ethnicity, or other socially relevant groupings	All participants are adults from China.
Population characteristics	All participants are adults from China. Supplementary Table 6 lists the demographic and clinical characteristics of the 80 colorectal cancer patients and 80 healthy controls.
Recruitment	All colorectal cancer patients were pathologically diagnosed by two senior pathologists. The enrollment criteria for healthy control subjects were as follows: (1) the absence of benign or malignant tumors; (2) a qualified physical examination finding no dysfunction of vital organs and (3) normal renal function and without albuminuria. No self-selection bias was expected to be introduced.
Ethics oversight	This study was approved by the Ethics Committee of the Institute of Basic Medical Sciences, Chinese Academy of Medical Sciences (#047-2019) with an exemption of informed consent and was performed according to the Declaration of Helsinki Principles.

Note that full information on the approval of the study protocol must also be provided in the manuscript.

Field-specific reporting

Please select the one below that is the best fit for your research. If you are not sure, read the appropriate sections before making your selection.

☒ Life sciences ☐ Behavioural & social sciences ☐ Ecological, evolutionary & environmental sciences

For a reference copy of the document with all sections, see nature.com/documents/nr-reporting-summary-flat.pdf

Life sciences study design

All studies must disclose on these points even when the disclosure is negative.

Sample size	In this study, we carried out a comprehensive evaluation of urinary proteome across multiple LC-MS platforms including about 756 proteome experiments. First, we optimized the sample, chromatography, and mass spectrometry procedure, and a total of 36 urinary proteome data were collected. Second, we prepared a urine peptide QC sample and distributed aliquots to 20 LC-MS platforms, and a total of 160 urinary proteome data were collected from 20 LC-MS platforms. Third, we prepared benchmarking sample A and sample B, with mixed human urine/cell, yeast and E. coli peptides in specified distinct ratios. The samples were analyzed in triplicate on three LC-MS platforms, and a total of 36 proteome data were collected. Fourth, we collected a clinical cohort comprising 80 urine samples from colorectal cancer (CRC) patients and 80 samples from matched healthy controls (HC). LC-MS data collection for these 160 samples was conducted sequentially on Orbitrap Fusion Lumos, Exploris 480, and timsTOF Pro 2. QC samples were randomly analyzed during the collection process for systematic evaluation of reproducibility. In total, the three platforms generated 527 DIA experiments.
Data exclusions	In total, 6 colorectal cancer patients data and 2 healthy control data were excluded from the downstream analysis duo to the low performance of these experiments.
Replication	Urine peptide QC sample data collected from 20 LC-MS platforms were analyzed in triplicate (technical replicates), and benchmarking samples data were acquired in triplicate (technical replicates). The experiments for colorectal cancer (CRC) patients and healthy controls (HC) samples were performed independently once. QC samples were randomly analyzed during the collection process for systematic evaluation of reproducibility.

Randomization	The acquisition of 80 colorectal cancer (CRC) patients and 80 healthy controls (HC) samples was randomized to avoid bias.
Blinding	The investigators who performed proteomics sample preparation and data acquisition were not blinded to clinical patient parameters as randomization in proteomics data generation was anyway performed to avoid systematic bias during the measurement and blinding is thus not relevant.

Reporting for specific materials, systems and methods

We require information from authors about some types of materials, experimental systems and methods used in many studies. Here, indicate whether each material, system or method listed is relevant to your study. If you are not sure if a list item applies to your research, read the appropriate section before selecting a response.

Materials & experimental systems

n/a	Involved in the study
☒	☐ Antibodies
☐	☒ Eukaryotic cell lines
☒	☐ Palaeontology and archaeology
☒	☐ Animals and other organisms
☒	☐ Clinical data
☒	☐ Dual use research of concern
☒	☐ Plants

Methods

n/a	Involved in the study
☒	☐ ChIP-seq
☒	☐ Flow cytometry
☒	☐ MRI-based neuroimaging

Eukaryotic cell lines

Policy information about cell lines and Sex and Gender in Research

Cell line source(s)	HEK 293 and Saccharomyces cerevisiae cell lines were purchased from ATCC.
Authentication	We relied on the authentication performed by ATCC and did not perform additional authentication.
Mycoplasma contamination	The cells were not tested for mycoplasma contamination.
Commonly misidentified lines (See ICLAC register)	No commonly misidentified lines were used in our study.

Plants

Seed stocks	N/A
Novel plant genotypes	N/A
Authentication	N/A
