# Supplementary material for: Standard operating procedure combined with comprehensive quality control system for multiple LC-MS platforms urinary proteomics
Source: Nat Commun. 2025 Jan 26;16:1051. doi: 10.1038/s41467-025-56337-4 (PMC11770173; doi:10.1038/s41467-025-56337-4)
Supplement: Supplementary file 3 — Description of Additional Supplementary files [file 41467_2025_56337_MOESM3_ESM.pdf]

File Name: Supplementary Data 1

Description: The detailed description of 58 intra-experiment metrics (A) and 23 interexperiment metrics (B) for quality control, and the detailed information reported from Spectronaut software (C).

File Name: Supplementary Data 2

Description: The overview of LC-MS conditions(A) for 20 LC-MS platforms, and the detailed LC-MS parameters for Thermo Orbitrap (B), Bruker timsTOF (C) and SCIEX ZenoTOF (D) data from 20 LC-MS platforms datasets.

File Name: Supplementary Data 3

Description: The Identified protein groups of 20 LC-MS platforms without LC-SOP (A) and with LC-SOP (B) from Spectronaut 18.0.

File Name: Supplementary Data 4

Description: The summary of metrics values and metrics scores report from MSCohort of 20 LC-MS platforms.

File Name: Supplementary Data 5

Description: Protein groups quantification results of benchmark samples (Fold change, p value, and adjusted p value were performed using the LIMMA package in R with all retained protein groups for each of these proteins had to satisfy a minimum number of 3 across replicated analyses).

File Name: Supplementary Data 6

Description: Clinical information of colorectal cancer (CRC) and healthy control (HC) samples used in this study. All summary data are medians ± interquartile range (IQR) with proportions.

File Name: Supplementary Data 7

Description: The three urine-specific quality marker panels to assess the degree of contamination of the samples, including erythrocytes (Geyer et al., 2019), cells and cellular debris (Winter et al., 2021), and serum contaminants (see Methods).

File Name: Supplementary Data 8

Description: Differentially expressed proteins in CRC and HC. (Fold change, p value, and adjusted p value were performed using the LIMMA package (version 3.58) in R (version 4.3) with the expectation that proteins significantly altered between CRC and HC exhibited Benjamini & Hochberg-adjusted $p < 0.05$).

File Name: Supplementary Data 9

Description: IPA Canonical pathway analysis and bio function analysis of CRC/HC related differentially expressed proteins.

File Name: Supplementary Data 10

Description: Proteomic model performance evaluation for CRC/HC stratification.
